# Histidine-Rich C-Terminal Tail of Mycobacterial GroEL1 and Its Copper Complex—The Impact of Point Mutations

**DOI:** 10.1021/acs.inorgchem.2c04486

**Published:** 2023-04-24

**Authors:** Anna Rola, Oscar Palacios, Merce Capdevila, Daniela Valensin, Elżbieta Gumienna-Kontecka, Sławomir Potocki

**Affiliations:** † Faculty of Chemistry, University of Wroclaw, 50-383 Wroclaw, Poland; ‡ Departament de Química, Universitat Autònoma de Barcelona, 08193 Cerdanyola del Vallès, Spain; § Department of Biotechnology, Chemistry and Pharmacy, University of Siena, Via A. Moro 2, 53100 Siena, Italy

## Abstract

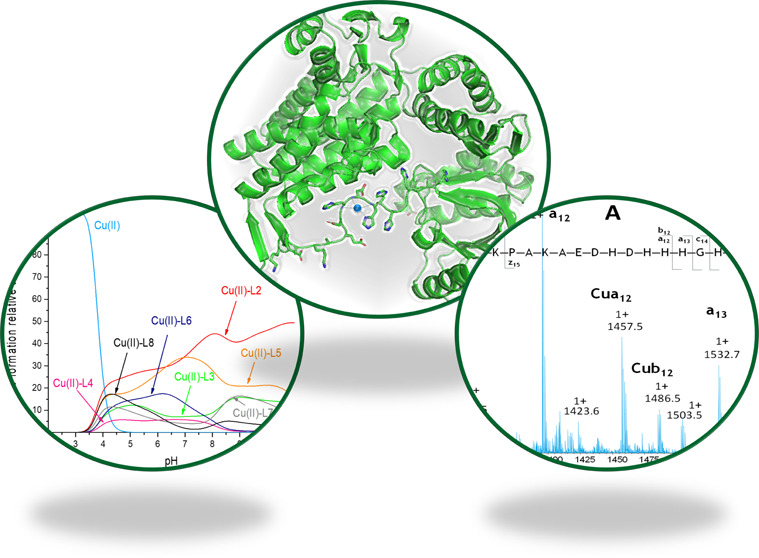

The mycobacterial histidine-rich GroEL1 protein differs significantly compared to the well-known methionine/glycine-rich GroEL chaperonin. It was predicted that mycobacterial GroEL1 can play a significant role in the metal homeostasis of *Mycobacteria* but not, as its analogue, in protein folding. In this paper, we present the properties of the GroEL1 His-rich C-terminus as a ligand for Cu(II) ions. We studied the stoichiometry, stability, and spectroscopic features of copper complexes of the eight model peptides: L1—Ac-DHDHHHGHAH, L2—Ac-DKPAKAEDHDHHHGHAH, and six mutants of L2 in the pH range of 2–11. We revealed the impact of adjacent residues to the His-rich fragment on the complex stability: the presence of Lys and Asp residues significantly increases the stability of the system. The impact of His mutations was also examined: surprisingly, the exchange of each single His to the Gln residue did not disrupt the ability of the ligand to provide three binding sites for Cu(II) ions. Despite the most possible preference of the Cu(II) ion for the His9–His13 residues (Ac-DKPAKAED**H**D**HHH-**) of the model peptide, especially the His11 residue, the study shows that there is not only one possible binding mode for Cu(II). The significance of this phenomenon is very important for the GroEL1 function—if the single mutation occurs naturally, the protein would be still able to interact with the metal ion.

## Abstract 2

The histidine-rich C-terminus (HRCT) of mycobacterial GroEL1 seems to be involved in Cu(II) homeostasis. We studied the coordination properties of model peptides: Ac-DKPAKAEDHDHHHGHAH and its six mutants in which one His residue is replaced with a Gln residue. The His9−His13 fragment has the highest affinity for Cu(II), and most probably, His11 is the main binding site for the metal ion. Interestingly, His residue rearrangement also impacts the formation of binuclear species.

## Introduction

Infections caused by *Mycobacterium* species remain a huge problem for the modern world with the rapidly growing number of multidrug-resistant (MDR) bacteria.^1−4^ The WHO report reveals that the infection caused by *Mycobacterium tuberculosis* is the most difficult to cure.^5^ In 2020, an estimated 1.3 million patients died due to tuberculosis and approximately 9.9 million people fell ill with the disease worldwide. *M. tuberculosis* survives in the host organism by penetrating the macrophages and manipulating their metal cation trafficking. The host organism defends itself from *M. tuberculosis*, e.g., by increasing copper and zinc concentrations in phagosomes.^6^ In order to develop a new method of treatment, it is necessary to understand the mechanisms of metal homeostasis in *M. tuberculosis*. More detailed studies on the role of important mycobacterial virulence factors are required to achieve this goal.

**Figure 1:**
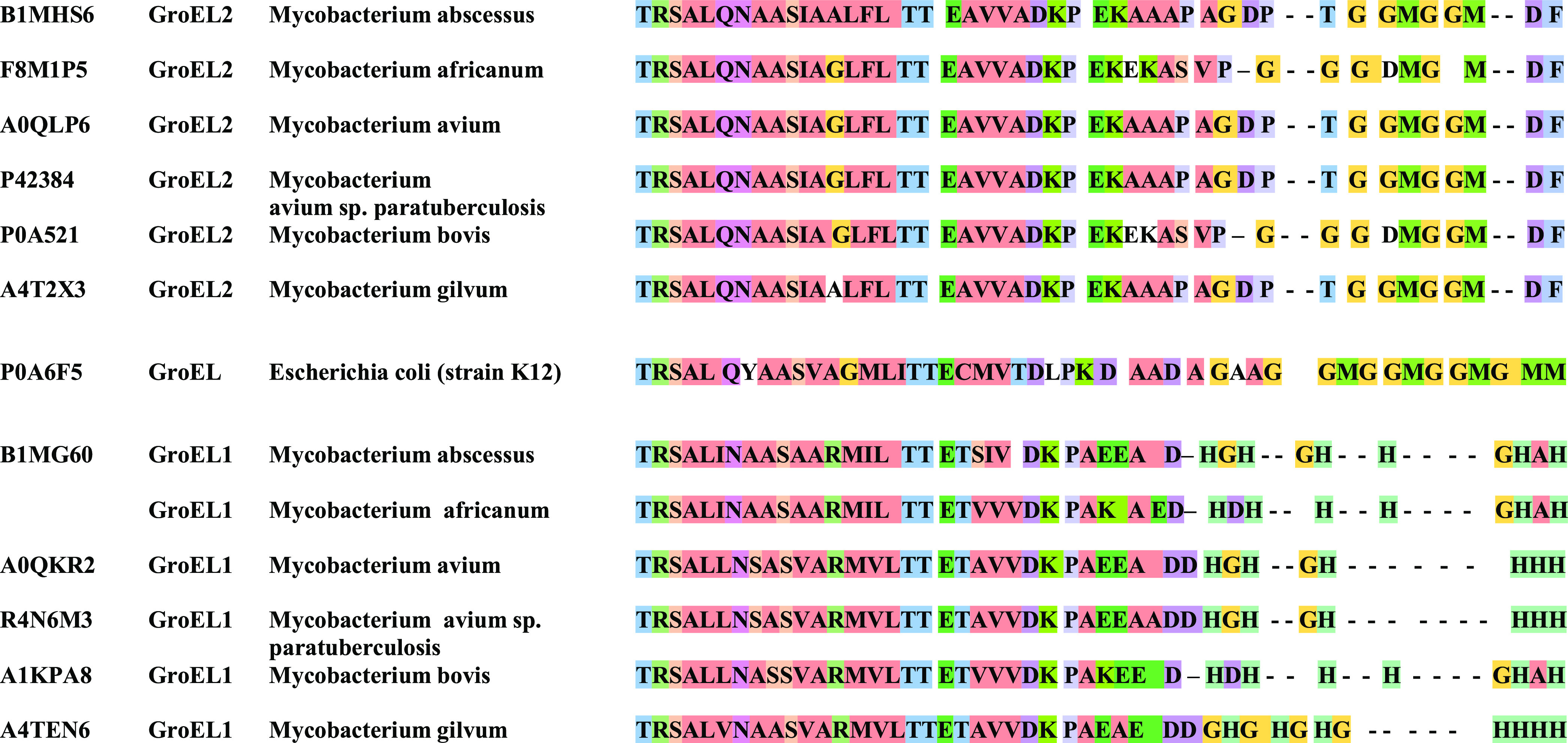
C-terminal sequence comparison of mycobacterial GroELs with the *E. coli* homologue. It suggests the existence of two protein families. The first shares a glycine–rich domain with the *E. coli* GroEL. The second possesses a distinct histidine-rich sequence.^13^

Chaperonins containing a histidine-rich C-terminal (HRCT) tail are potentially significant factors of maintaining metal homeostasis in *Mycobacteria*. In this paper, we investigate a particular type of mycobacterial histidine-rich chaperonins—GroEL1. The GroEL1 participates in metabolic and energetic adaptation under stress.^7^ The well-known *Escherichia coli* GroEL has the ability to oligomerize in two heptameric structures stacked back to back, in order to promote protein folding supported by the co-chaperonin GroES, in a manner dependent on ATP.^8^ The mycobacterial GroEL1 histidine-rich protein exists only as a monomer, even in the presence of stabilizers of oligomeric forms.^8,9^ It has been discovered that Cu(II) induces a response of the mycobacterial GroEL1 (*M. tuberculosis*) and its histidine-rich C-terminal region plays an essential role in this process.^46^ The histidine-rich motif is responsible for metal ion trafficking in a variety of organisms, including bacteria.^10−12^ It was predicted that proteins containing such specific sequences may be used as a molecular target for a new generation of medicines.^13^ The GroEL1 protein containing the HRCT tail cannot exert the same biological function as well-studied, typical GroEL proteins containing the methionine- and glycine-rich C-terminus (Figure 1).^13^ Therefore, a unique role played by the HRCT tail of mycobacterial GroEL1 in maintaining metal homeostasis is very probable. With the use of the UniProtBlast database, we have identified a large number of metal-transporting/binding proteins derived from different bacterial and fungal organisms, containing histidine-rich motifs, of which sequences are 100% identical with the motifs occurring in different GroEL1 proteins. The **H**D**HHH**G**H**A**H** motif from *M. tuberculosis* GroEL1 (UniProt Code: P9WPE9)^14^ is also present, e.g., in copper-transporting ATPase protein (UniProt Code: Q2K000, *Rhizobium etli*),^15^ metal tolerance protein 12 (UniProt Code: W9RBJ6, *Morus notabilis*),^16^ and nickel/cobalt efflux system (UniProt Code: A4Z171, *Bradyrhizobium* sp.).^17^

The results of this bioinformatic research confirm that peptides containing histidine-rich motifs may indeed be involved in maintaining metal homeostasis. In this paper, we analyze the properties of the Cu(II)–HRCT systems from the coordination chemistry point of view. With a variety of methods, we examined the stability and stoichiometry of the formed complexes as well as their geometry and number or characteristic of metal-binding sites. We have chosen two model peptides as ligands for metal ions: L1: Ac-D**H**D**HHH**G**H**A**H** and L2: Ac-DKPAKAED**H**D**HHH**G**H**A**H**. L1 is a HRCT fragment of *M. tuberculosis* GroEL1 (UniProt kode: P9WPE9), and L2 is a longer fragment of the same protein. This approach allowed us to analyze the impact adjacent to histidine-rich fragment amino acids on the properties of complexes formed by these ligands with divalent metal ions. Studies on the longer sequence have one more advantage: because of the high flexibility of the histidine-rich domain of GroEL1, three-dimensional (3D) structures of mycobacterial GroEL1 obtained so far by X-ray diffraction (XRD) crystallography research lack approximately 20 amino acids on the C-terminal tail.^13^ We also studied six mutants of L2, in which one His residue is replaced with a Gln residue (L3: Ac-DKPAKAED**Q**D**HHH**G**H**A**H**, L4: Ac-DKPAKAED**H**D**QHH**G**H**A**H**, L5: Ac-DKPAKAED**H**D**HQH**G**H**A**H**, L6: Ac-DKPAKAED**H**D**HHQ**G**H**A**H**, L7: Ac-DKPAKAED**H**D**HHH**G**Q**A**H**, L8: Ac-DKPAKAED**H**D**HHH**G**H**A**Q)** to investigate the impact of each of the His residues on the Cu(II) complex properties.

## Experimental Section

### Materials

All peptide ligands were purchased from KareBay Biochem, Inc. (certified purity: L1 = 98.27%, L2 = 98.08%, L3 = 98.52%, L4 = 99.12%, L5 = 98.96%, L6 = 98.19%, L7 = 98.32%, L8 = 98.12%). The identity of the peptides was evaluated based on mass spectrometry. The purity was checked based on potentiometric titrations using the Gran method.^18^ The solutions of metal ions were prepared using Cu(ClO_4_)_2_·6H_2_O (Merck, HPLC grade) and filtered, using double-distilled water. The concentration of the stock solution was periodically checked via ICP. All solvents were prepared with freshly doubly distilled water. For the preparation of peptide and complex solutions, 4 × 10^–3^ M HClO_4_ (Merck) acid was used. The ionic strength was adjusted to 0.1 M by adding NaClO_4_ (Merck).

### Mass Spectrometry Measurements

The high-resolution mass spectra of all samples were obtained by means of electrospray ionization time-of-flight mass spectrometry (ESI-TOF MS) using a Micro TOF-Q instrument (Bruker Daltonics, Bremen, Germany) interfaced to a Series 1200 HPLC Agilent pump and controlled using Compass software. ESI-L low-concentration tuning mix (Agilent Technologies, Santa Clara, CA) was used as a calibrator. A 5:95 mixture of acetonitrile:ammonium acetate (15 mM) was used as a running buffer for neutral (pH 7.5) conditions. Instrument conditions were as follows: 10–45 μL of sample solution was injected through a polyether heteroketone (PEEK) tube (0.5–1.5 m, 0.18 mm i.d.) at 25–50 μL·min^–1^, applying a capillary counter-electrode voltage of 3.5–5. 5 kV, a dry temperature of 90–110 °C, dry gas at 6 L min^–1^, and a spectral collection range of 300–2000 *m*/*z*. All spectra were processed using Bruker Data Analysis software. The metal-to-ligand molar ratio was 1:1.

### Potentiometric Measurements

Stability constants for the proton and Cu(II) complexes were calculated from the pH-metric titration curves, in the pH range of 2.5–11 at 298 K and an ionic strength of 0.1 M NaClO_4_. All the experiments were performed under an argon atmosphere, to protect the sample from carbonate appearance. The measurements were carried out using a Dosimat 665 Methrom titrator connected to a Methrom 691 pH meter equipped with a pH electrode InLab Semi-Micro (Mettler Toledo). The thermostabilized glass cell was equipped with a microburette delivery tube, a magnetic stirring system, and an inlet–outlet tube for argon. 0.1 M carbonate-free NaOH was used as a titrant. The electrodes were calibrated daily for hydrogen ion concentration by titrating HClO_4_ with NaOH under the same experimental conditions as mentioned above. The Gran method allowed us to determine the purity and the exact concentration of the ligand solutions.^18^ The peptide concentration was 0.5 mM, and the Cu(II)-to-ligand molar ratio was 0.9:1.

Stability constant calculations were performed using HYPERQUAD 2006 software.^19^ The reported log β values refer to the overall equilibria
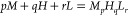
1
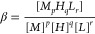
2where charges are omitted for clarity; log *K*_step_ values refer to the protonation process
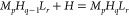
3

(charges omitted; p might also be 0). Standard deviations were calculated using HYPERQUAD 2006 and refer to random errors only. The speciation, K_d_ values, and competition diagrams were obtained with the HYSS program.^20^

### CD, UV–vis, and EPR Spectroscopy Measurements

The absorption spectra were recorded on a Jasco V-750 (Jasco) spectrophotometer, and the circular dichroism (CD) spectra were obtained on a Jasco J-1500 spectrometer at 298 K in the 750–250 nm range. For the experiments, the total sample volume of 2.5 mL was used. The parameters of the instruments were as followed: scanning speed: 200 nm/min, data pitch: 0.5 nm, and number of accumulations: 3. The concentration of the peptides was 0.5 mM, and the complex samples were prepared by adding metal ions to the peptide solution. The molar ratio of metal to ligand were 0.9:1. Data were processed using Origin 2016.

For the secondary structure studies, the parameters of the CD spectrometer were changed as follows: scanning speed: 100 nm/min, data pitch: 0.5 nm, number of accumulations: 6, and range: 280–180 nm. The optical pathway for these experiments was 0.1 cm.

Electron paramagnetic resonance (EPR) spectra were recorded at an X-band frequency (9.5 GHz) at 77 K on a Bruker ELEXSYS E500 CW–EPR spectrometer equipped with an ER 036TM NMR teslameter and E41 FC frequency counter. The complex solutions were prepared in an aqueous solution containing 4 mM HClO_4_ and 0.1 M NaClO_4_, with 25% ethylene glycol as a cryoprotectant. The concentration of Cu(II) was 1 mM with 0.9:1 metal-to-peptide molar ratio. The pH was adjusted with HClO_4_ and NaOH solutions. The EPR parameters were obtained from computer simulations of the experimental spectra using Bruker’s WIN-EPR SIMFONIA software, version 1.2 (Billerica). All spectra were drawn in Origin 2016.

## Results

### Protonation Equilibria of Ac-DHDHHHGHAH (L1), Ac-DKPAKAEDHDHHHGHAH (L2), and Mutants of the Latter

Protonation constants of the examined peptides and probable assignments to the particular chemical groups are presented in Table 1. The peptides were protected at the N-terminus. Ac-DHDHHHGHAH (L1) behaves like H_9_L acid, Ac-DKPAKAEDHDHHHGHAH (L2) behaves like H_13_L acid, and L2 mutants (L3–L8) exhibit 11 protonation constants in the pH range of 2.5–11.

**Table 1:** Protonation Constants of L1–L8 Peptides at *T* = 298 K and *I* = 0.1 M (NaClO_4_) and Potential Assignments to Appropriate Side Chains/Chemical Groups^a^^,^^b^

species	log β*_jk_*^c^	p*K*_a_^d^	residue	species	log β*_jk_*^c^		p*K*_a_^d^		residue	
L1: Ac-DHDHHHGHAH				L2: Ac-DKPAKAEDHDHHHGHAH						
[HL]^3–^	7.83 (1)	7.83	His	[HL]^5–^		10.48 (1)	10.48		Lys	
[H_2_L]^2−^	15.10 (1)	7.28	His	[H_2_L]^4–^		20.24 (1)	9.76		Lys	
[H_3_L]^−^	21.96 (1)	6.86	His	[H_3_L]^3–^		28.35 (1)	8.11		His	
[H_4_L]	28.38 (1)	6.42	His	[H_4_L]^2–^		35.68 (1)	7.33		His	
[H_5_L]^+^	34.44 (1)	6.06	His	[H_5_L]^−^		42.75 (1)	7.07		His	
[H_6_L]^2+^	39.88 (1)	5.44	His	[H_6_L]		49.25 (1)	6.50		His	
[H_7_L]^3+^	43.69 (2)	3.81	Asp	[H_7_L]^+^		55.46 (1)	6.21		His	
[H_8_L]^4+^	46.77 (2)	3.08	Asp	[H_8_L]^2+^		60.95 (1)	5.49		His	
[H_9_L]^5+^	49.27 (2)	2.49	C-terminus	[H_9_L]^3+^		65.22 (1)	4.27		Glu	
				[H_10_L]^4+^		69.11 (2)	3.89		Asp	
				[H_11_L]^5+^		72.40 (2)	3.29		Asp	
				[H_12_L]^6+^		75.30 (2)	2.90		Asp	
				[H_13_L]^7+^		77.83 (2)	2.53		C-terminus	
L3: Ac-DKPAKAEDQDHHHGHAH				L4: Ac-DKPAKAEDHDQHHGHAH						
[HL]^5–^	10.82 (1)	10.82	Lys	[HL]^5–^		11.01 (1)	11.01		Lys	
[H_2_L]^4–^	20.74 (1)	9.92	Lys	[H_2_L]^4–^		20.96 (1)	9.95		Lys	
[H_3_L]^3–^	28.59 (2)	7.84	His	[H_3_L]^3–^		28.74 (1)	7.78		His	
[H_4_L]^2–^	35.81 (1)	7.23	His	[H_4_L]^2–^		35.98 (1)	7.24		His	
[H_5_L]^−^	42.68 (1)	6.86	His	[H_5_L]^−^		42.83 (1)	6.85		His	
[H_6_L]	48.98 (1)	6.30	His	[H_6_L]		49.17 (1)	6.35		His	
[H_7_L]^+^	55.96 (1)	5.98	His	[H_7_L]^+^		55.17 (1)	6.00		His	
[H_8_L]^2+^	59.71 (2)	4.74	Glu	[H_8_L]^2+^		59.88 (2)	4.71		Glu	
[H_9_L]^3+^	63.78 (2)	4.07	Asp	[H_9_L]^3+^		63.94 (3)	4.05		Asp	
[H_10_L]^4+^	67.34 (3)	3.56	Asp	[H_10_L]^4+^		67.55 (3)	3.61		Asp	
[H_11_L]^5+^	70.79 (2)	3.45	Asp	[H_11_L]^5+^		70.97 (2)	3.42		Asp	
L5: Ac-DKPAKAEDHDHQHGHAH				L6: Ac-DKPAKAEDHDHHQGHAH						
[HL]^5–^	11.11 (1)	11.11	Lys	[HL]^5–^		11.02 (1)	11.02		Lys	
[H_2_L]^4–^	21.26 (1)	10.16	Lys	[H_2_L]^4–^		21.01 (1)	9.98		Lys	
[H_3_L]^3–^	29.13 (1)	7.87	His	[H_3_L]^3–^		28.87 (2)	7.86		His	
[H_4_L]^2–^	36.29 (1)	7.16	His	[H_4_L]^2–^		36.10 (2)	7.23		His	
[H_5_L]^−^	43.07 (1)	6.78	His	[H_5_L]^−^		43.07 (2)	6.97		His	
[H_6_L]	49.36 (1)	6.30	His	[H_6_L]		49.42 (2)	6.35		His	
[H_7_L]^+^	55.10 (1)	5.74	His	[H_7_L]^+^		55.59 (2)	6.17		His	
[H_8_L]^2+^	59.46 (1)	4.36	Glu	[H_8_L]^2+^		60.54 (2)	4.96		Glu	
[H_9_L]^3+^	63.36 (2)	3.90	Asp	[H_9_L]^3+^		64.63 (3)	4.08		Asp	
[H_10_L]^4+^	66.73 (2)	3.37	Asp	[H_10_L]^4+^		68.27 (4)	3.65		Asp	
[H_11_L]^5+^	69.78 (2)	3.06	Asp	[H_11_L]^5+^		71.82 (2)	3.55		Asp	
L7: Ac-DKPAKAEDHDHHHGQAH				L8: Ac-DKPAKAEDHDHHHGHAQ						
[HL]^5–^	11.10 (1)	11.10	Lys	[HL]^5–^		11.06 (2)		11.06		Lys
[H_2_L]^4–^	21.11 (1)	10.01	Lys	[H_2_L]^4–^		21.45 (1)		10.39		Lys
[H_3_L]^3–^	28.95 (1)	7.84	His	[H_3_L]^3–^		29.54 (3)		8.09		His
[H_4_L]^2–^	36.15 (1)	7.20	His	[H_4_L]^2–^		36.49 (3)		6.95		His
[H_5_L]^−^	42.98 (1)	6.83	His	[H_5_L]^−^		43.39 (3)		6.89		His
[H_6_L]	49.27 (1)	6.29	His	[H_6_L]		49.51 (3)		6.12		His
[H_7_L]^+^	55.06 (1)	5.79	His	[H_7_L]^+^		55.31 (3)		5.80		His
[H_8_L]^2+^	59.51 (1)	4.45	Glu	[H_8_L]^2+^		59.75 (3)		4.44		Glu
[H_9_L]^3+^	63.44 (2)	3.93	Asp	[H_9_L]^3+^		63.73 (3)		3.98		Asp
[H_10_L]^4+^	66.77 (3)	3.33	Asp	[H_10_L]^4+^		67.04 (4)		3.31		Asp
[H_11_L]^5+^	70.02 (2)	3.25	Asp	[H_11_L]^5+^		70.34 (3)		3.30		Asp

a The ligand concentration was 0.0005 M. The Cu(II)-to-ligand molar ratio was 0.9:1. *I* = 0.1 M NaClO_4_ and *T* = 298 K.

b The standard deviations are reported in parentheses as uncertainties on the last significant figure.

c Protonation constants are presented as cumulative log β*_jk_* values. Standard deviations of the last digits are given in parentheses, at the values obtained directly from the experiment. L stands for a peptide with acid–base active groups. β(H*_j_*L*_k_*) = [H*_j_*L*_k_*]/([H]*_j_*[L]*^k^*), in which [L] is the concentration of the fully deprotonated peptide.

d p*K*_a_ = log β(H*_j_*L*_k_*) – log β(H_*j*–1_L*_k_*).

Potentiometric measurements detected nine protonation constants for the L1 peptide (H_9_L). The first p*K*_a_ value corresponds to the deprotonation of the C-terminus. The next two p*K*_a_ values arise from the deprotonation of the carboxyl groups of the two aspartic acids. Another six p*K*_a_ values can be assigned to the deprotonations of the imidazole groups of the six histidine residues.

The L2 can be considered the H_13_L ligand. The first p*K*_a_ value arises from the deprotonation of the C-terminus. The next three p*K*_a_ values correspond to the deprotonation of the three carboxylic side chain groups of aspartic acid residues, while the following one corresponds to the deprotonation of the glutamic acid side chain group. The next six p*K*_a_ values can be assigned to the deprotonation of histidine imidazole groups, and the last two p*K*_a_ values arise from the deprotonation of two lysine residues.

The L3–L8 behave like H_11_L ligands. The first three p*K*_a_ values arise from the deprotonation of the three carboxylic side chain groups of aspartic acid residues, the following one corresponds to the deprotonation of the glutamic acid side chain group, and the next five correspond to the deprotonation of histidine residues. The last two p*K*_a_ values arise from the deprotonation of two lysine residues.

The exact p*K*_a_ values of all ligands are listed in Table 1. p*K*_a_ constant values of His residue deprotonation are similar to those found in other similar His-rich systems.^21−23^ In the case of all ligands, at acidic pH, the formation of highly positively charged species occurs, e.g., [H_10_L]^4+^, [H_9_L]^5+^, [H_12_L]^6+^, and [H_13_L]^7+^. It is caused by the presence of 5–6 His and 0–2 Lys residues. These species correspond to the deprotonation of Asp residues or the C-terminus. p*K*_a_ values of these steps (2.90–3.65) are lower than the p*K*_a_ value of the free Asp residue (3.86), and the peptide charge can be an explanation for it. Highly positively charged species tend to favor the deprotonation, resulting in the charge decrease, which contributes to the system stability.^24,25^ In the case of [H_9_L]^3+^, the p*K*_a_ constant values (3.89–4.08) also corresponding to the Asp residue deprotonation are more like the p*K*_a_ value of the free Asp residue because the charge of this species is not highly positive anymore.

### Cu(II) Complexes

**Figure 2:**
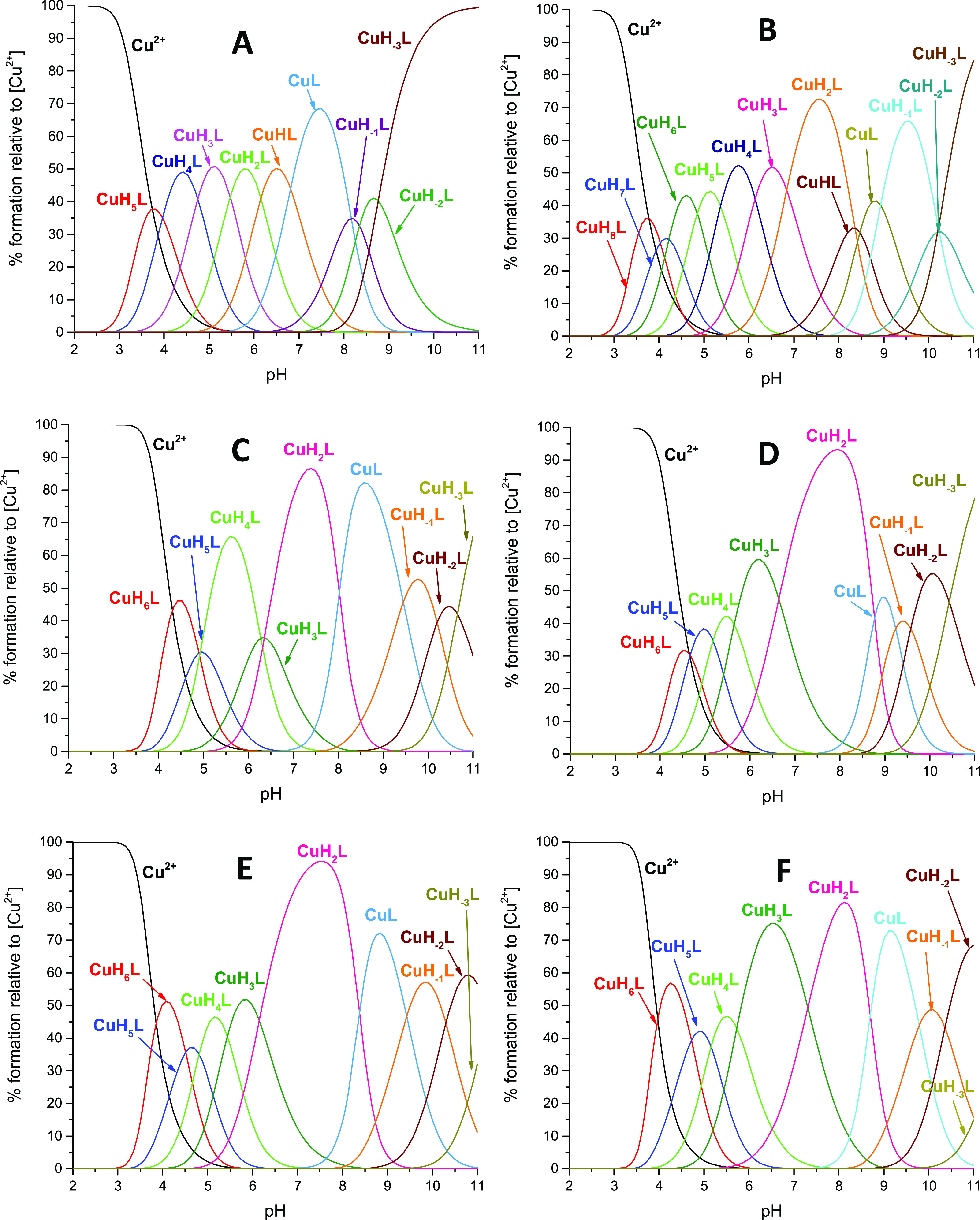

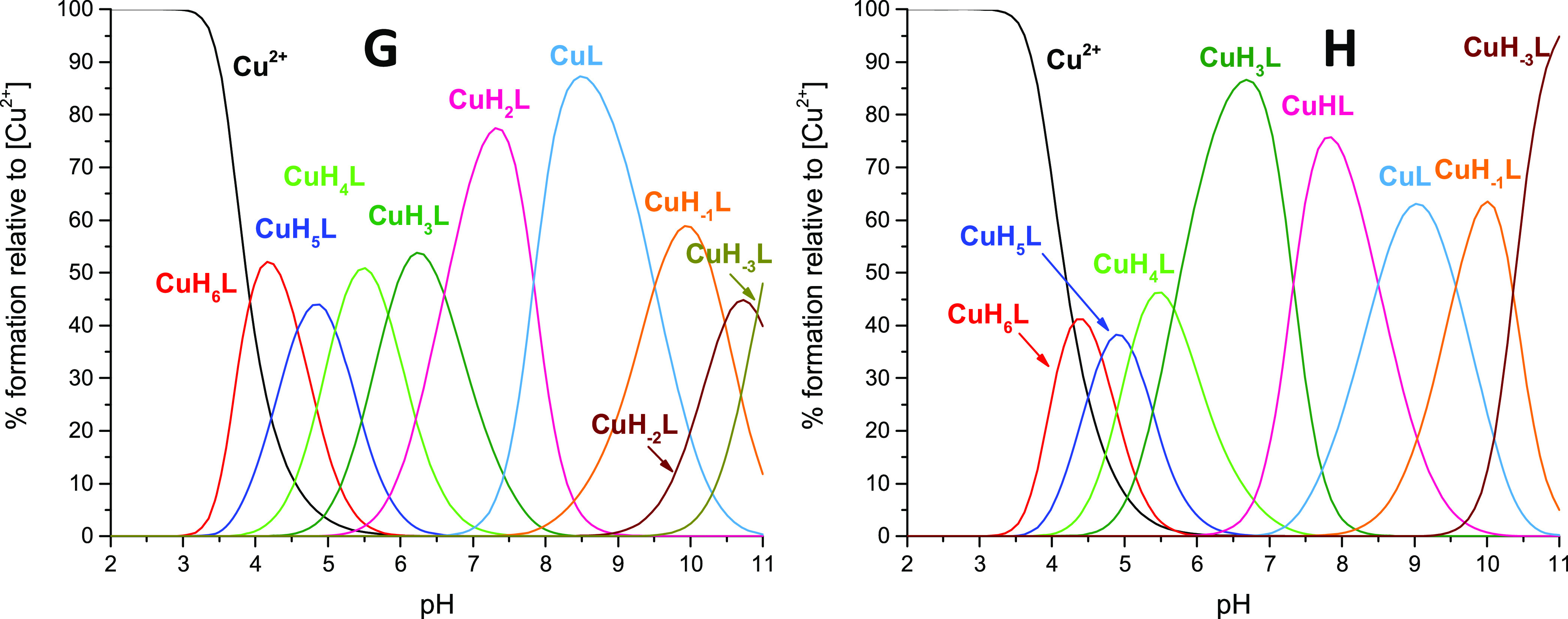
Distribution diagram of complex forms in the Cu(II)-L systems. L—Ac-DHDHHHGHAH (A), Ac-DKPAKAEDHDHHHGHAH (B), Ac-DKPAKAEDQDHHHGHAH (C), Ac-DKPAKAEDHDQHHGHAH (D), Ac-DKPAKAEDHDHQHGHAH (E), Ac-DKPAKAEDHDHHQGHAH (F), Ac-DKPAKAEDHDHHHGQAH (G), and Ac-DKPAKAEDHDHHHGHAQ (H). M/L = 1:1. pH range: 2–11.

The presence of Cu(II) complexes with the model peptides was determined by a variety of methods. Signals in the mass spectra have been assigned to ions of ligands or Cu(II)–L complexes. ESI-MS peak assignments were based on the comparison between the precisely calculated and experimental *m*/*z* values and their isotopic patterns. To establish the number and chemical characteristic of metal binding sites as well as the geometry and stability of the complexes, potentiometric titrations, EPR, CD, and UV–Vis spectroscopy have been used. Stability constants for the studied complexes are collected in Table 2; the distribution diagrams are presented in Figure 2, and MS/UV–Vis/EPR/CD spectra are presented in the Supporting Information. Table S1 shows the major coordination modes of the Cu(II)-L system at a pH range of 4.0–11.0, where L means the appropriate model peptide.

**Table 2:** Potentiometric Data for Cu(II)-L1–Cu(II)-L8 Complexes in an Aqueous Solution of HClO_4_ at *I* = 0.1 M (NaClO_4_), [Cu(II)] = 0.00045 M, *T* = 298 K (of Potentiometric Titration); Molar Ratio M/L—0.9:1

species	log β*_ijk_*^a^	p*K*_a_^b^	donor set	species	log β*_ijk_*^a^	p*K*_a_^b^	donor set
Cu(II)-Ac-DHDHHHGHAH (L1)				Cu(II)-Ac-DKPAKAEDHDHHHGHAH (L2)			
[CuH_–3_L]^4–^	–13.47 (4)	8.76	{1N_im_. 3N^–^}	[CuH_–3_L]^6–^	–13.26 (3)	10.20	{1N_im_. 3N^–^}
[CuH_–2_L]^3–^	–4.71 (2)	8.35	{2N_im_. 2N^–^}	[CuH_–2_L]^5–^	–3.06 (3)	10.18	{2N_im_. 2N^–^}
[CuH_–1_L]^2–^	3.64 (2)	8.17	{2N_im_. 1N^–^}	[CuH_–1_L]^4–^	7.12 (3)	8.99	{3N_im_. 1N^–^}
[CuL]^−^	11.81 (2)	6.81	{2N_im_}	[CuL]^3–^	16.10 (4)	8.46	{3N_im_}
[CuHL]	18.62 (2)	6.16	{2N_im_}	[CuHL]^2–^	24.57 (2)	8.36	{3N_im_}
[CuH_2_L]^+^	24.78 (2)	5.47	{2N_im_}	[CuH_2_L]^−^	32.93 (2)	6.84	{3N_im_}
[CuH_3_L]^2+^	30.25 (4)	4.75	{2N_im_}	[CuH_3_L]	39.77 (3)	6.14	{3N_im_}
[CuH_4_L]^3+^	34.99 (3)	3.97	{2N_im_}	[CuH_4_L]^+^	45.91 (3)	5.37	{3N_im_}
[CuH_5_L]^4+^	38.96 (3)		{1N_im_}	[CuH_5_L]^2+^	51.28 (3)	4.85	{2N_im_}
				[CuH_6_L]^3+^	56.13 (4)	4.23	{2N_im_}
				[CuH_7_L]^4+^	60.35 (7)	4.03	{1N_im_}
				[CuH_8_L]^5+^	64.38 (8)		{1N_im_}
Cu(II)-Ac-DKPAKAEDQDHHHGHAH (L3)				Cu(II)-Ac-DKPAKAEDHDQHHGHAH (L4)			
[CuH_–3_L]^6–^	–14.05 (10)	10.65	{1N_im_. 3N^–^}	[CuH_–3_L]^6–^	–14.49 (5)	10.43	{1N_im_. 3N^–^}
[CuH_–2_L]^5–^	–3.40 (10)	10.20	{1N_im_. 3N^–^}	[CuH_–2_L]^5–^	–4.06 (5)	9.58	{1N_im_. 3N^–^}
[CuH_–1_L]^4–^	6.80 (10)	9.45	{2N_im_. 2N^–^}	[CuH_–1_L]^4–^	5.52 (5)	9.24	{2N_im_. 2N^–^}
[CuL]^3–^	16.25 (9)		{3N_im_. 1N^–^}	[CuL]^3–^	14.76 (5)		{2N_im_. 2N^–^}
[CuH_2_L]^−^	32.30 (8)	6.36	{3N_im_}	[CuH_2_L]^−^	32.30 (4)	6.63	{3N_im_}
[CuH_3_L]	38.66 (10)	6.28	{2N_im_}	[CuH_3_L]	38.94 (3)	5.65	{2N_im_}
[CuH_4_L]^+^	44.94 (8)	4.93	{2N_im_}	[CuH_4_L]^+^	44.59 (5)	5.18	{2N_im_}
[CuH_5_L]^2+^	49.87 (10)	4.67	{2N_im_}	[CuH_5_L]^2+^	49.77 (4)	4.67	{2N_im_}
[CuH_6_L]^3+^	54.77 (10)		{1N_im_}	[CuH_6_L]^3+^	54.44 (6)		{1N_im_}
Cu(II)-Ac-DKPAKAEDHDHQHGHAH (L5)				Cu(II)-Ac-DKPAKAEDHDHHQGHAH (L6)			
[CuH_–3_L]^6–^	–14.10 (3)	11.25	{1N_im_. 3N^–^}	[CuH_–3_L]^6–^	–16.68 (3)	11.65	{1N_im_. 3N^–^}
[CuH_–2_L]^5–^	–2.85 (3)	10.30	{1N_im_. 3N^–^}	[CuH_–2_L]^5–^	–5.02 (3)	10.36	{1N_im_. 3N^–^}
[CuH_–1_L]^4–^	7.45 (3)	9.43	{2N_im_. 2N^–^}	[CuH_–1_L]^4–^	5.34 (3)	9.80	{2N_im_. 2N^–^}
[CuL]^3–^	16.88 (2)		{3N_im_. 1N^–^}	[CuL]^3–^	15.13 (3)		{3N_im_. 1N^–^}
[CuH_2_L]^−^	33.64 (2)	6.15	{3N_im_}	[CuH_2_L]^−^	32.53 (3)	7.31	{3N_im_}
[CuH_3_L]	39.79 (2)	5.44	{2N_im_}	[CuH_3_L]	39.84 (2)	5.72	{3N_im_}
[CuH_4_L]^+^	45.23 (2)	4.81	{2N_im_}	[CuH_4_L]^+^	45.57 (2)	5.17	{2N_im_}
[CuH_5_L]^2+^	50.04 (3)	4.52	{2N_im_}	[CuH_5_L]^2+^	50.73 (2)	4.71	{2N_im_}
[CuH_6_L]^3+^	54.55 (3)		{1N_im_}	[CuH_6_L]^3+^	55.45 (2)		{1N_im_}
Cu(II)-Ac-DKPAKAEDHDHHHGQAH (L7)				Cu(II)-Ac-DKPAKAEDHDHHHGHAQ (L8)			
[CuH_–3_L]^6–^	–14.25 (4)	10.92	{1N_im_. 3N^–^}	[CuH_–3_L]^6–^	–13.99 (4)		{1N_im_. 3N^–^}
[CuH_–2_L]^5–^	–3.33 (3)	10.46	{1N_im_. 3N^–^}	[CuH_–1_L]^4–^	–6.73 (4)	9.57	{1N_im_. 3N^–^}
[CuH_–1_L]^4–^	7.14 (4)	9.51	{2N_im_. 2N^–^}	[CuL]^3–^	16.30 (4)	8.51	{2N_im_. 2N^–^}
[CuL]^3–^	16.65 (3)		{3N_im_. 1N^–^}	[CuHL]^2–^	24.80 (3)		{3N_im_. 1N^–^}
[CuH_2_L]^−^	32.32 (2)	6.60	{3N_im_}	[CuH_3_L]	39.46 (2)	5.68	{3N_im_}
[CuH_3_L]	38.92 (2)	5.83	{2N_im_}	[CuH_4_L]^+^	45.14 (3)	5.10	{2N_im_}
[CuH_4_L]^+^	44.75 (3)	5.10	{2N_im_}	[CuH_5_L]^2+^	50.24 (3)	4.68	{2N_im_}
[CuH_5_L]^2+^	49.85 (3)	4.58	{2N_im_}	[CuH_6_L]^3+^	54.92 (3)		{1N_im_}
[CuH_6_L]^3+^	54.43 (3)		{1N_im_}				

a Cu(II) stability constants are presented as cumulative log β*_ijk_* values. L stands for a fully deprotonated peptide ligand that binds Cu(II). Standard deviations of the last digits are given in parentheses at the values obtained directly from the experiment. β(M*_i_*H*_j_*L*_k_*) = [M*_i_*H*_j_*L*_k_*]/([M]*_i_*[H]*_j_*[L]*_k_*), where [L] is the concentration of the fully deprotonated peptide.

b p*K*_a_ = log β(M*_i_*H*_j_*L*_k_*) – log β(M*_i_*H_*j*–1_L*_k_*).

Electrospray ionization mass spectrometry revealed the stoichiometry of the studied complexes. In the spectrum of the Cu(II)-L1 system (equimolar mixture), two main types of ions were detected, [L]^+^ (*m*/*z* = 1241.52, *z* = +1) and [CuL]^+^ (*m*/*z* = 1302.44, *z* = +1), corresponding to the single-charged ion of the ligand and its Cu(II) complex, respectively (Figure S1). In the spectra of Cu(II) and L2 equimolar mixture (Figure S2A) also, two main types of ions were observed: [L]^2+^ (*z* = 2+, *m*/*z* = 991.44) and [CuL]^2+^ (*z* = 2+, *m*/*z* = 1022.40). They correspond to the double-charged ion of the ligand and its Cu(II) complex, respectively. In addition, we observed a weak signal corresponding to [Cu_2_L]^2+^. However, the intensity of this signal is not significant compared to that of [CuL]^2+^. In the case of the equimolar mixtures of Cu(II) and mutants (L3–L8), the same types of ions as for Cu(II)-L2 were detected. The spectra of all mutants were identical, and an example of a spectrum is presented in Figure S3A. The 1: 1 metal-to-ligand interaction in equimolar mixture is confirmed by potentiometric calculations. The comparison of the simulated and experimental isotopic distribution of [CuL]^+^ and [CuL]^2+^ confirmed the identity of signals at *m*/*z* = 1302.44 for Cu(II)-L1, 1022.40 for Cu(II)-L2, and 1017.43 for Cu(II)-L3–Cu(II)-L8.

In the presence of an excess of metal ions, the intensity of [Cu_2_L]^2+^ ions increases in the case of the studied systems Cu(II)-L2–Cu(II)-L8 (Figures S2B, S3B, S4), but the abundance ratio of [CuL]^2+^/[Cu_2_L]^2+^ differs between the particular systems. It confirms that the studied peptides could bind more than one copper ion per molecule when an excess of copper is applied. Unfortunately, due to the problems with complex peptide solubility that commonly occur in the high concentration of metal ions, we could not obtain reliable results of potentiometric and spectroscopic (UV–Vis, CD) titrations.

MS studies provide interesting results concerning the potential role of particular His residues in Cu(II) binding and the role of the HRCT itself. The intensity of the MS signals does not always correlate with the concentration, as the ability of the molecule to be ionized is also important in terms of intensity. Here, we compare the signals of two very similar species with the same charge—[CuL]^2+^ and [Cu_2_L]^2+^—so we can assume that their ionization should be very similar, and consequently, the relative intensity could give information about the abundance of these particular forms. The intensity ratio of [CuL]^2+^/[Cu_2_L]^2+^ = 2:1, approximately, in the case of Cu(II)-Ac-DKPAKAEDHDHHHGHAH (L2) (Figure S2B). In the case of Cu(II)-Ac-DKPAKAEDHD**Q**HHGHAH (L4), the intensity ratio [CuL]^2+^/[Cu_2_L]^2+^ is the biggest and equal to approximately 6:1 (Figure S4A). In the case of Cu(II)-Ac-DKPAKAED**Q**DHHHGHAH (L3) and Cu(II)-Ac-DKPAKAEDHDH**Q**HGHAH (L5) systems, the intensity ratio [CuL] ^2+^/[Cu_2_L]^2+^ is close to 1:1 (Figures S3B and S4B). The reverse correlation is observed in the case of Cu(II)-Ac-DKPAKAEDHDHH**Q**GHAH (L6), Cu(II)-Ac-DKPAKAEDHDHHHG**Q**AH (L7), and Cu(II)-Ac-DKPAKAEDHDHHHGHA**Q** (L8) (Figure S4C–E); the intensity ratio is reversed in favor of binuclear species and equal to 1:6, 1:4, and 1:7, respectively. The results obtained suggest that the ability to form binuclear species depends on the rearrangement of His residues.

In the case of Cu(II)-L1, His binding starts when the charge is +4. The deprotonation of a second, metal binding His residue is followed by the change of charge to +3. The p*K*_a_ of [CuH_4_L]^3+^ is low compared to the p*K*_a_ values of the free ligand related to the deprotonation of the His residue: 5.44, 6.06, 6.42, 6.86, 7.28, and 7.83 (Table 2). The low value of [CuH_4_L]^3+^ p*K*_a_ is a result of a relatively high positive charge.^24,25^ With the decrease of the charge, the p*K*_a_ values of particular His residues increase. Amide nitrogen deprotonation starts when the complex is relatively highly negatively charged (−3) and is forced by the basic pH.

In the case of Cu(II)-L2, the first His residue deprotonation starts when the molecule is more positively charged than Cu(II)-L1 (+5), due to the presence of two Lys residues. Another three deprotonations followed by the change of charge to +2 and most probably are related to the deprotonation of one Glu and two His residues. p*K*_a_ values of [CuH_7_L]^4+^, [CuH_6_L]^3+^, and [CuH_5_L]^2+^ are decreased compared to the p*K*_a_ of Glu (4.27) and His residues in the free ligand: 5.49, 6.21, 6.50, 7.07, and 7.33 (Table 2). This is due to the increase of the charge in the presence of the Cu(II) ion.^24,25^ p*K*_a_ values of three next species, [CuH_4_L]^+^, [CuH_3_L], and [CuH_2_L]^−^, related to the deprotonation of another three His residues, are higher due to the decrease of the charge. Amide nitrogen deprotonations are forced by the basic pH and cause the further decrease of the charge to a highly negative value: −6.

Cu(II)-L3–Cu(II)-L8 systems are of lower charge at acidic pH compared to Cu(II)-L2, due to the lack of one His residue. The general correlation between the decreased p*K*_a_ values related to His residue deprotonation and highly positive charge of species is preserved. The details of the Cu(II)-L1–Cu(II)-L8 systems are described in further sections.

#### Cu(II)-L1

The potentiometric titrations of the Cu(II)-L1 system revealed the existence of nine complex forms at the pH range of 2.5–11: [CuH_5_L]^4+^, [CuH_4_L]^3+^, [CuH_3_L]^2+^, [CuH_2_L]^+^, [CuHL], and [CuL], and [CuH_–1_L]^2–^, [CuH_–2_L]^3–^, and [CuH_–3_L]^4–^. L1 peptide starts to bind Cu(II) ions at pH above 3 (Figure 2A). The first complex species is [CuH_5_L]^4+^ with a maximum concentration at pH 4.0, and most probably, it comes from the deprotonation of the first His residue. Asp residues are also deprotonated in this form. The presence of a band with a maximum absorption at 720 nm in the UV–vis spectra suggests one nitrogen atom in the Cu(II) ion coordination sphere (Figure S5A).^21,26^ EPR parameters for the complex at pH 4.05 (*A* = 160.5 G, *g*_∥_ = 2.31, Figure S5B) also confirm the interactions of Cu(II) with one nitrogen atom and suggest a {1N_im_} coordination mode for [CuH_5_L]^4+^.^21^ The next species, [CuH_4_L]^3+^ (maximum concentration at pH 4.5), is formed as a result of the deprotonation of the second His residue. The p*K*_a_ value of 3.97 for this step probably corresponds to p*K*_a_ = 7.83 for one His residue in the free ligand and is significantly reduced, suggesting the presence of other His side chains in the coordination sphere of Cu(II). Above pH 4.0, a significant blue shift (720–600 nm) is observed in the UV–vis spectra, suggesting the formation of a complex with a second or third nitrogen atom bond (Figure S5A).^21,26^ EPR parameters for the complex at pH 5.07 (*A* = 176.5 G, *g*_∥_ = 2.27, Figure S5B) confirm the interaction of Cu(II) with two nitrogen atoms.^21^ The other four species, [CuH_3_L]^2+^, [CuH_2_L]^+^, [CuHL], and [CuL]^−^, with maximum concentrations at pH 5.0, 6.0, 6.5, and 7.5, respectively, arise from the deprotonation of four, probably, non-metal-binding His residues. p*K*_a_ values of 4.75, 5.47, 6.16, and 6.81 for these steps could probably correspond to p*K*_a_ = 5.44, 6.06, 6.42, and 7.28, respectively, for His residues in the free ligand. However, this assignment is only approximate—we cannot precisely indicate which p*K*_a_ corresponds to the particular His residue deprotonation. It is obvious that the adjacent residues and their acceptor/donor abilities as well as the overall complex charges impact the scheme of deprotonation and p*K*_a_ values. They are not reduced in the presence of Cu(II), suggesting that four His residues do not bind Cu(II). Under the conditions of the UV–vis/CD experiment, the complex at pH 6.00–8.01 was not soluble. However, the EPR parameters for the complex (*A* = 180.0 G, *g*_∥_ = 2.26 at pH 6.12 and *A* = 175.3 G, *g*_∥_ = 2.25 at pH 7.03) allowed us to establish the {2N_im_} coordination mode for [CuH_3_L]^2+^, [CuH_2_L], [CuHL]^−^, and [CuL]^2–^ (Figure S5B).^21^ No significant changes in the UV–vis spectrum were observed at pH 8.01, what supports the above-mentioned result. [CuH_–1_L]^2–^, [CuH_–2_L]^3–^, and [CuH_–3_L]^4–^ forms most probably arise from the deprotonation of three amide nitrogen atoms (maximum concentration at around pH 8.0, 9.0, and 10.0, respectively). The blue shift (600 to 540 nm) at pH 9.11 observed in the UV–vis and the changes in the EPR spectra at pH 9.07 (*A* = 196.0 and *g*_∥_ = 2.21) indicate that the third nitrogen atom, arising from the deprotonation of the first amide bond, coordinates with Cu(II) (Figures 2A and S5A,B).^21,26,27^ This finding is supported by the presence of the d–d band at 500 and 620 nm (negative and positive Cotton effect), corresponding to the copper(II)–amide nitrogen interactions in peptide complexes (Figure S5C).^28−31^ A further increase of pH causes the increase of the d–d band in the CD spectrum, a slight blue shift in the UV–vis spectrum, and the change of EPR spectral parameters (*A* = 194.0 and *g*_∥_ = 2.19).^21,32^ It confirms the involvement of a second and third amide nitrogen atom in Cu(II) binding, replacement of two N_im_ with the N^–^ atom in the Cu(II) coordination sphere, and formation of a complex with a square planar geometry. The coordination mode of [CuH_–1_L]^2–^, [CuH_–2_L]^3–^, and [CuH_–3_L]^4–^ is {2N_im_. 1N^–^}, {2N_im_. 2N^–^}, and {1N_im_. 3N^–^}, respectively.

#### Cu(II)-L2

The potentiometric titrations of the Cu(II)-L2 system revealed the existence of 12 complex forms at the pH range of 2–11: [CuH_8_L]^5+^, [CuH_7_L]^4+^, [CuH_6_L]^3+^, [CuH_5_L]^2+^, [CuH_4_L]^+^, [CuH_3_L], [CuH_2_L]^−^, [CuHL]^2–^, and [CuL]^3–^ and [CuH_–1_L]^4–^, [CuH_–2_L]^5–^, and [CuH_–3_L]^6–^. Similar to L1, L2 starts to bind Cu(II) ions at pH above 3.0 (Figure 2B). [CuH_8_L]^5+^ and [CuH_7_L]^4+^, with a maximum concentration at around pH 4.0, most probably come from the deprotonation of a His and Glu residue, respectively. Asp residues are also deprotonated in this form. In the UV–vis spectra at pH 3.99, we can observe a band with a maximum absorption at 720 nm, suggesting that one nitrogen atom is in the metal ion coordination sphere (Figure S6A).^21,26^ EPR parameters for the complex at pH 4.02 (*A* = 163.3 G, *g*_∥_ = 2.31, Figure S6B) indicate the interactions of Cu(II) with one nitrogen atom, suggesting a {1N_im_} coordination mode for [CuH_7_L]^4+^ and [CuH_8_L]^5+^.^21^ The next two species, [CuH_6_L]^3+^ and [CuH_5_L]^2+^, (maximum concentrations at pH 4.5 and 5.0, respectively) could be formed as a result of the deprotonation of a second and third His residue. The p*K*_a_ values of 4.23 and 4.85 most probably correspond to p*K*_a_ = 7.34 and 5.49, respectively, for His residues in the free ligand. Only one of these values is significantly reduced in the presence of Cu(II), suggesting that a second His imidazole ring is in the coordination sphere of the metal ion. At pH 5.08, a significant blue shift (720–600 nm) is observed in the UV–vis spectra, confirming the involvement of an additional nitrogen atom in Cu(II) binding (Figure S6A).^21,26^ EPR parameters for the complex at pH 5.11 (*A* = 177.1 G, *g*_∥_ = 2.27, Figure S6B) also support the interaction of Cu(II) with two nitrogen atoms.^21^ A {2N_im_} coordination mode is therefore assigned to the [CuH_6_L]^3+^ and [CuH_5_L]^2+^ species. [CuH_4_L]^+^, with a maximum concentration at pH 6.0, could arise from the deprotonation of a fourth His residue. The p*K*_a_ value of 5.37 for this step could probably correspond to p*K*_a_ = 8.11 for His residue in the free ligand and is significantly reduced. EPR parameters for the complex at pH 6.12 (*A* = 179.1 G. *g*_∥_ = 2.26. Figure S6B) confirm the interaction of Cu(II) with three nitrogen atoms, suggesting a {3N_im_} coordination mode for [CuH_4_L]^+^.^21,22,33,34^ Another two species, [CuH_3_L] and [CuH_2_L]^−^ (maximum at pH 6.5 and 7.5, respectively), most likely come from the deprotonation of two non-metal-binding His residues. The p*K*_a_ values of 6.14 and 6.84 for these steps could probably correspond to p*K*_a_ = 6.50 and 7.07, respectively, for the His residue in the free ligand—they are not significantly reduced. No significant changes in the UV–vis, EPR, and CD spectra were observed at pH 6.07–9.09, confirming the above-mentioned result. [CuHL]^2–^, [CuL]^3–^, and [CuH_–3_L]^6–^ presumably come from the deprotonation of three amide nitrogen atoms (maximum concentration at around pH 8.0, 9.0, and 10.0, respectively), and [CuH_–1_L]^4–^ and [CuH_–2_L]^5–^ (maximum concentrations at around pH 9.5 and 10.2, respectively) could arise from the deprotonation of two Lys residues that are not involved in Cu(II) binding. The EPR parameters at pH 9.01 (*A* = 193.2 and *g*_∥_ = 2.21) suggest that the formation of a complex with a fourth nitrogen atom bond started (Figure S6B).^21,32^ Additionally, this finding is supported by the presence of the d–d band at 500 and 620 nm (negative and positive Cotton effect) at pH 9.09, corresponding to the copper(II)–amide nitrogen interactions in peptide complexes (Figure S6C).^28−31^ A further increase of pH to 10.0 and 11.0 causes the increase of the d–d band in the CD spectra and the shift of a band in the UV–vis spectra (Figure S6A,C).^21,26,28−31^ It confirms the involvement of a second and third amide nitrogen atom in Cu(II) binding, replacement of two N_im_ with a N^–^ atom in the Cu(II) coordination sphere, and formation of a complex with a square planar geometry. The final UV–vis (λ_max_ = 525 nm) and EPR (*A* = 196.9, *g*_∥_ = 2.19) set of parameters at pH 11.04 (λ_max_ = 530 nm) strongly confirms the interaction of Cu(II) with four nitrogen atoms (Figure S6A,B).^21,26,32^ The coordination modes of [CuHL]^2–^, [CuL]^3–^, and [CuH_–1_L]^4–^ are {3N_im_. 1N^–^}. For [CuH_–2_L]^5–^ and [CuH_–3_L]^6–^, the coordination mode would be {2N_im_. 2N^–^} and {1N_im_, 3N^–^}, respectively.

#### Cu(II)-L3

The potentiometric titrations of the Cu(II)-L3 system revealed the existence of nine complex forms at the pH range of 2–11: [CuH_6_L]^3+^, [CuH_5_L]^2+^, [CuH_4_L]^+^, [CuH_3_L], [CuH_2_L]^−^, and [CuL]^3–^ and [CuH_–1_L]^4–^, [CuH_–2_L]^5–^, and [CuH_–3_L]^6–^. Similar to L1 and L2, L3 peptide starts to bind Cu(II) ions at pH above 3.0 (Figure 2C). [CuH_6_L]^3+^ with a maximum concentration at pH 4.5 most probably comes from the deprotonation of the first His side chain. Asp and Glu residues are also deprotonated in this form. The arise of a band with a maximum absorption at 690 nm in the UV–vis spectrum (pH 4.16) indicates that one nitrogen atom is involved in Cu(II) binding (Figure S7A).^21,26^ Additionally, the EPR parameters (*A* = 163.0, *g*_∥_ = 2.30) of the Cu(II)-L3 system at pH around 4.0 support this observation, confirming a {1N_im_} coordination mode for [CuH_6_L]^3+^ (Figure S7B).^21^ The next species [CuH_5_L]^2+^ with a maximum at a pH of around 5.0 presumably appears in the solution as a result of the second His residue deprotonation. The p*K*_a_ value of 4.67 could correspond to p*K*_a_ = 7.23 for one of the His residues in the free ligand and is significantly reduced, suggesting that another imidazole nitrogen atom is involved in Cu(II) binding. The shift of a band in the UV–vis spectrum at pH 5.15 (690 to 630 nm) and EPR parameters (*A* = 173.4, *g*_∥_ = 2.27) suggest the interaction of two nitrogen atoms with Cu(II) (Figure S7A,B).^21,26^ It indicates a {2N_im_} coordination mode for [CuH_5_L]^2+^. The species [CuH_4_L]^+^ and [CuH_3_L] with a maximum concentration at pH 5.5 and 6.5, respectively, could come from the deprotonation of the third and fourth His residues. The p*K*_a_ values of 4.93 and 6.28 probably correspond to p*K*_a_ = 5.98 and 6.83, respectively, for two of the His residues in the free ligand and are not significantly reduced, suggesting the non-metal binding characteristic of these residues. The shift of a band in the UV–vis spectrum (630 to 600 nm) along with the EPR spectral parameters (*A* = 180.5, *g*_∥_ = 2.25) at pH around 6.0 and 7.0 suggests that there is an equilibrium between forms in which two or three nitrogen atoms bind Cu(II) (Figure S7A,B).^21,22,26^ It is related to the formation of another species, [CuH_2_L]^−^—it presumably comes from the deprotonation of the fifth His imidazole ring. The p*K*_a_ value of 6.36 could probably correspond to p*K*_a_ = 7.84 for one of the His residues in the free ligand and is reduced, supporting the metal binding characteristic of this residue. The shift of a band in the UV–vis spectrum (600 to 580 nm) and the change of EPR parameters (*A* = 181.3, *g*_∥_ = 2.24) at pH around 8.0 finally indicate that three nitrogen atoms bind Cu(II) (Figure S7A,B).^21,22,26,34^ The coordination mode for [CuH_2_L]^−^ is {3N_im_}. [CuL]^3–^ species with a maximum concentration at pH 8.5 is most likely related to the deprotonation of one amide bond and one non-metal binding under the conditions of the experimental Lys residue. Arising of a d–d band at pH 8.11–9.27 in the CD spectrum and a shift in the UV–vis spectrum (580 to 550 nm) confirm the interactions between Cu(II) and amide nitrogen (Figure S7C).^28−31^ The coordination mode for [CuL]^3–^ is therefore {3N_im_,N^–^}. [CuH_–1_L]^4–^ and [CuH_–2_L]^5–^, with a maximum concentration at pH 10.0 and 10.5, respectively, appear in the solution most probably as a result of the second and third amide bond deprotonation. The changes of EPR parameters (*A* = 189.9, *g*_∥_ = 2.20) and appearance of a blue shift in the UV–vis spectrum (550 to 530 nm) at pH around 10 confirm the existence of forms in which only four and not three nitrogen atoms bind Cu(II) (Figure S7A,B).^21,26,32^ In the CD spectrum, a d–d band at 500 nm (negative Cotton effect) and at 620 nm (positive Cotton effect) increases, which confirms the interaction of amide nitrogens with Cu(II) and formation of a complex with a square planar geometry (Figure S7C).^28−31^ No further changes were observed when the pH increases. It indicates that [CuH_–3_L]^6–^ comes from the deprotonation of the second non-metal-binding Lys residue and that the coordination mode for [CuH_–1_L]^4–^ is {2N_im_, 2N^–^} and for [CuH_–2_L]^5–^/[CuH_–3_L]^6–^, it is {N_im_, 3N^–^}.

#### Cu(II)-L4

The potentiometric titrations of the Cu(II)-L4 system indicate that nine complex forms exist at the pH range of 2–11: [CuH_6_L]^3+^, [CuH_5_L]^2+^, [CuH_4_L]^+^, [CuH_3_L], [CuH_2_L]^−^, and [CuL]^3–^ and [CuH_–1_L]^4–^, [CuH_–2_L]^5–^, and [CuH_–3_L]^6–^ (Figure 2D). The first form [CuH_6_L]^3+^ (maximum concentration at pH 4.0) most probably appears as a result of the first His deprotonation. In the UV–vis spectrum at pH 4.12, we can observe a band with a maximum at around 700 nm, and the parameters of the EPR spectrum at pH 4.10 are as follows: *A* = 161.0 and *g*_∥_ = 2.31 (Figure S8A,B). Both phenomena indicate that one nitrogen atom is in the coordination sphere of Cu(II). The coordination mode for [CuH_6_L]^3+^ is therefore {1N_im_}.^21,26^ The next species [CuH_5_L]^2+^ (maximum concentration at pH 5.0) most likely comes from the second His residue deprotonation. The p*K*_a_ value of 4.67 for this step could probably correspond to p*K*_a_ = 7.24 for one of the His residues in the free ligand and is significantly reduced, suggesting that another imidazole nitrogen atom is involved in Cu(II) binding. This result is confirmed by the presence of a blue shift in the UV–vis spectrum (700 to 630 nm) and the change of EPR parameters (*A* = 171.9, *g*_∥_ = 2.27) (Figure S8A,B).^21,26^ {2N_im_} can be therefore assigned to [CuH_5_L]^2+^. Another two species, [CuH_4_L]^+^ and [CuH_3_L] (maximum concentration at pH 5.5 and 6.0, respectively), are probably formed as a result of the third and fourth His imidazole group deprotonation that most probably are not involved in Cu(II) binding. The p*K*_a_ values of 5.18 and 5.65 for these steps could be related to p*K*_a_ = 6.00 and 6.35, respectively, for two of the His residues in the free ligand and are not significantly reduced. The small blue shift in the UV–vis (630 to 600 nm) and EPR parameters (*A* = 181.3, *g*_∥_ = 2.25) at pH around 6.0 and 7.0 (Figure S8A,B) suggest that there could be an equilibrium of species in which two or three nitrogen atoms are present in the coordination sphere of the metal ion.^21,26^ A similar situation was observed in the case of the copper complex of the first mutant—L3. This is in agreement with the potentiometric results (Figure 2D)—at pH around 6.0 and 7.0, another species starts to form, [CuH_2_L]^−^ (with a maximum concentration at pH 8.0). It most likely comes from the deprotonation of the fifth His imidazole group. The p*K*_a_ value of 6.63 for this step could probably correspond to p*K*_a_ = 7.78 for one of the His residues in the free ligand and is reduced, suggesting that another imidazole nitrogen atom is involved in Cu(II) binding. This is confirmed by the presence of a blue shift in the UV–vis spectrum (600 to 580 nm) and the change of the EPR parameters (*A* = 183.6, *g*_∥_ = 2.24, Figure S8A,B).^21,26,33,34^ The coordination mode for [CuH_4_L]^+^ and [CuH_3_L] is {2N_im_}, and for [CuH_2_L]^−^, it is {3N_im_}. The next species [CuL]^3–^ (maximum concentration at pH 9.0) comes from the deprotonation of two amide nitrogen atoms. The appearance of a blue shift at pH 9.10 along with the EPR parameter set (*A* = 195.3, *g*_∥_ = 2.21) indicates that another nitrogen atom is involved in metal binding (Figure S8A,B).^21,26,32^ Additionally, the d–d band at 500 and 600 nm (negative and positive Cotton effect), characteristic for Cu(II) complexes of amide nitrogen, supports this finding (Figure S8C).^28−31^ It indicates a {2N_im_, 2N^–^} coordination mode for [CuL]^3–^. [CuH_–1_L]^4–^ (maximum concentration at pH 10.0) most probably arises from the deprotonation of a non-metal-binding Lys residue, while [CuH_–2_L]^5–^ (maximum concentration at pH 10.0) is formed as a result of the third amide nitrogen deprotonation. The increase of pH to 10.11 causes the increase of the CD band at 500 and 600 nm and a further blue shift in the UV–Vis spectrum (580 to 540 nm), which supports this finding (Figure S8A,C).^21,26,28−31^ The further increase of pH does not significantly affect the spectra. This is in agreement with the potentiometric data, since [CuH_–3_L]^6–^ that dominates at pH 11.0 is most probably related to the second non-metal-binding Lys residue deprotonation. The coordination mode for [CuH_–3_L]^6–^ should be {1N_im_, 3N^–^}.

#### Cu(II)-L5

The potentiometric titrations of the Cu(II)-L5 system revealed that nine exact complex forms exist at the pH range of 2–11: [CuH_6_L]^3+^, [CuH_5_L]^2+^, [CuH_4_L]^+^, [CuH_3_L], [CuH_2_L]^−^, and [CuL]^3–^ and [CuH_–1_L]^4–^, [CuH_–2_L]^5–^, and [CuH_–3_L]^6–^ (Figure 2E). The first species [CuH_6_L]^3+^ with a maximum concentration at pH 4.0 most probably arises from the deprotonation of the first His imidazole group. In the UV–vis spectrum at pH 4.09, we can observe a band with a maximum at around 690 nm that corresponds to the d–d band characteristic for a copper complex with one nitrogen atom (Figure S9A).^21,26^ This observation is supported by the EPR spectrum parameters for the Cu(II)-L5 system at pH 4.19 (*A* = 161.0, *g*_∥_ = 2.31) (Figure S9B), suggesting a {1N_im_} coordination mode for [CuH_6_L]^3+^.^21^ Another species, [CuH_5_L]^2+^ (maximum concentration at pH 4.8), most likely comes from the deprotonation of another His residue. The p*K*_a_ value of 4.52 for this step could correspond to p*K*_a_ = 7.16 for one of the His residues in the free ligand and is significantly reduced, suggesting that a second imidazole nitrogen atom is in the Cu(II) coordination sphere. This is confirmed by the presence of a blue shift in the UV–vis spectra (690 to 640 nm) and the change of EPR parameters (*A* = 171.9, *g*_∥_ = 2.27) at pH around 5.0 (Figure S9A,B).^21,26^ Therefore, the {2N_im_} coordination mode can be assigned to [CuH_5_L]^2+^. [CuH_4_L]^+^ and [CuH_3_L] (maximum concentration at pH 5.3 and 6.0, respectively) species presumably arise from the deprotonation of another two His imidazole groups. The p*K*_a_ values of 4.81 and 5.44 for these steps could probably correspond to p*K*_a_ = 5.74 and 6.30 for two of the His residues in the free ligand and are not significantly reduced, suggesting that these His imidazole nitrogen atoms do not bind Cu(II). The increase of pH to 6.13 causes a small shift of a band in the UV–vis spectra (640 to 600 nm) and the change of EPR parameters (*A* = 181.3, *g*_∥_ = 2.25) (Figure S9A,B).^21,22,26,34^ It suggests the existence of an equilibrium of species in which two and three nitrogen atoms interact with Cu(II)—this is in agreement with the potentiometric results and related to the arise of another species—[CuH_2_L]^−^ (Figure 2E). [CuH_2_L]^−^, the most abundant form with a maximum concentration at pH 7.4, most probably comes from the deprotonation of the fifth His residue. The p*K*_a_ value of 6.15 for this step could correspond to p*K*_a_ = 7.87 for one of the His residues in the free ligand and is significantly reduced, suggesting that a third imidazole nitrogen atom binds Cu(II). In the UV–vis spectrum at pH 7.13 (and 8.17), we can observe a blue shift (600 to 580 nm), and in the EPR spectrum, a shift of parameters was detected (*A* = 183.6, *g*_∥_ = 2.25 at pH 7.07 and *g*_∥_ = 2.24 at pH 8.06), confirming that a third His residue is present in the Cu(II) coordination sphere and that a coordination mode for this species should be {3N_im_} (Figure S9A,B).^21,22,26^ Another species, [CuL]^3–^ (maximum concentration at pH 9.0), most probably arises from the deprotonation of one amide nitrogen and one Lys residue. Three phenomena could support this suggestion: (1) the presence of a slight blue shift of a band in the UV–vis spectrum (580 to 570 nm) at pH 9.09 suggests that there is an equilibrium between species in which three and four nitrogen atoms are involved in Cu(II) binding; (2) EPR parameters at pH 9.12 change to *A* = 195.3 and *g*_∥_ = 2.21; and (3) in the CD spectrum at pH 9.09, the appearance of an abundant d–d band at 500 and 620 nm indicates that Cu(II) starts to form a bond with amide nitrogen (Figure S9A–C).^21,26,28−31^ A coordination mode for [CuL]^3–^ is {3N_im_, 1N^–^}. Two other species, [CuH_–1_L]^4–^ and [CuH_–2_L]^5–^ (maximum concentration at pH 10.0 and 11.0, respectively), presumably arise from the deprotonation of two other amide bonds that bind Cu(II). This finding is supported by the presence of a blue shift in the UV–vis spectra at pH 10.12 (570 to 540 nm), changes in EPR parameters (*A* = 194.5, *g*_∥_ = 2.20), and the increase of a d–d band at 500 and 620 nm in the CD spectrum (related to the the formation of the N^–^–Cu(II) bond) (Figure S9A–C).^21,26,28−32^ No significant changes were then observed with a further increase of the pH, which supports the idea that all amide bonds deprotonated before pH around 11.0 and that the last species [CuH_–3_L]^6–^ most likely comes from the deprotonation of a non-metal-binding Lys residue. Therefore, the coordination mode for [CuH_–1_L]^4–^, [CuH_–2_L]^5–^, and [CuH_–3_L]^6–^ is, respectively, {2N_im_, 2N^–^}, {1N_im_, 3N^–^}, and {1N_im_, 3N^–^}.

#### Cu(II)-L6

The potentiometric titrations of the Cu(II)-L6 system indicated that nine exact complex forms exist at the pH range of 2–11: [CuH_6_L]^3+^, [CuH_5_L]^2+^, [CuH_4_L]^+^, [CuH_3_L], [CuH_2_L]^−^, and [CuL]^3–^ and [CuH_–1_L]^4–^, [CuH_–2_L]^5–^, and [CuH_–3_L]^6–^. [CuH_6_L]^3+^ with a maximum at pH 4.3 most probably arises from the deprotonation of the first His imidazole group (Figure 2F). The involvement of this residue in metal binding is confirmed using two techniques: in the UV–vis spectrum at pH 4.09 (Figure S10A), the presence of a band with a maximum at 680 nm indicates that one imidazole nitrogen is in the coordination sphere of Cu(II) and the EPR parameters at pH 4.15 are in agreement with the UV–vis results (*A* = 161.4, *g*_∥_ = 2.30, Figure S10A,B).^21,26^ It suggests a {1N_im_} coordination mode for [CuH_6_L]^3+^. Another species, [CuH_5_L]^2+^ (maximum concentration at pH 5.0), most likely comes from the deprotonation of the second His residue. The p*K*_a_ value of 4.71 for this could correspond to p*K*_a_ = 6.97 for one of the His residues in the free ligand and is significantly reduced—it indicates that a second imidazole nitrogen atom binds Cu(II). The changes in the UV–vis and EPR spectra at pH around 5 (the presence of a blue shift: 680–615 nm, Figure S10A; *A* = 171.1, *g*_∥_ = 2.27, Figure S10B) confirm that two nitrogen atoms bind Cu(II) and that the {2N_im_} coordination mode can be assigned to [CuH_5_L]^2+^.^21,26^ Another species, [CuH_4_L]^+^ (maximum concentration at pH 5.5), most probably arises from the deprotonation of a non-binding His residue. The p*K*_a_ value of 5.17 for this step could correspond to p*K*_a_ = 6.17 for one of the His residues in the free ligand and is not significantly reduced, suggesting that this residue is not involved in Cu(II) binding. However, the changes in the UV–vis and EPR spectra at pH around 6.0 (the presence of a slight blue shift: 615–600 nm, Figure S10A; *A* = 174.4, *g*_∥_ = 2.26, Figure S10B) suggest the coexistence of two species in which two and three nitrogen atoms are in the coordination sphere, which is in agreement with the potentiometric data (Figure 2F).^21,22,26,33,34^ This is due to the formation of [CuH_3_L] (maximum concentration at pH 6.5)—a species that is most probably related to the deprotonation of the fourth His residue. The p*K*_a_ value of 5.72 for this step could correspond to p*K*_a_ = 7.23 for one of the His residues in the free ligand and is significantly reduced, suggesting that this residue strongly interacts with Cu(II). The increase of pH to 7.12 causes the appearance of a blue shift in the UV–vis spectrum (600 to 585 nm), and the EPR parameters at pH 7.07 change to *A* = 177.3 and *g*_∥_ = 2.25 (Figure S10A,B). It confirms that at pH around 9.0, the species in which three nitrogen atoms bind Cu(II) dominates.^21,22,26,33^ A coordination mode for [CuH_4_L]^+^ and [CuH_3_L] is {2N_im_} and {3N_im_}, respectively. [CuH_2_L]^−^ (maximum concentration at pH 8.0) most probably arises from the deprotonation of the fifth His residue. The p*K*_a_ value of 7.31 for this could be related to p*K*_a_ = 7.86 for one of the His residues in the free ligand and is not significantly reduced. It suggests that this residue does not bind Cu(II). At pH around 8.0, no significant changes in the UV–vis and EPR spectra were observed, which supports this assumption (Figure S10A,B).^21,26,32^ The next species [CuL]^3–^ (maximum concentration at pH 9.0) most probably corresponds to the deprotonation of one non-binding Lys residue and amide nitrogen. The slight changes in the UV–vis and EPR spectra at pH around 9.0 (the presence of a slight blue shift: 585–575 nm, Figure S10A; *A* = 195.4, *g*_∥_ = 2.21, Figure S10B) suggest the coexistence of a species in which three and four nitrogen atoms are in the coordination sphere. This is in agreement with the potentiometric data (Figure 2F).^21,22,26,32^ Additionally, in the CD spectrum at pH 9.09, we can observe the arising of a d–d band at 500 and 600 nm, commonly found in the spectra of Cu(II) complexes with an amide nitrogen atom (Figure S10C).^28−31^ The coordination mode for [CuL]^3–^ is {3N_im_, N^–^}. [CuH_–1_L]^4–^ reaches its maximum concentration at pH 10.0. The increase of pH to 10.10 causes a significant blue shift in the UV–vis spectrum (575–540 nm, Figure S10A), the change of EPR parameters (*A* = 198.9, *g*_∥_ = 2.21, Figure S10B), and the increase of a d–d band at 500 and 600 nm in the CD spectrum (Figure S10C), confirming the increasing abundance of a form in which four nitrogen atoms are present in the Cu(II) coordination sphere and another nitrogen atom deprotonates.^21,22,26,28−32^ The coordination mode for [CuH_–1_L]^4–^ is therefore {2N_im_, 2N^–^}. [CuH_–2_L]^5–^ and [CuH_–3_L]^6–^ (maximum concentration at pH 11.0 and above 11.0, respectively) species are most probably related to the deprotonation of the second Lys residue and third amide bond. The increase of pH to 11.10 causes the shift of a band in the UV–vis spectrum (540–520 nm, Figure S10A) and the change of EPR parameters (*A* = 191.4, *g*_∥_ = 2.20,Figure S10B), confirming that another amide nitrogen is in the Cu(II) coordination sphere.^21,26^ The [CuH_–2_L]^5–^ and [CuH_–3_L]^6–^ coordination mode is {N_im_, 3N^–^}.^32^

#### Cu(II)-L7

The potentiometric titrations of the Cu(II)-L7 system indicated that similar to Cu(II)-L4, Cu(II)-L5, and Cu(II)-L6, nine exact complex forms exist at the pH range of 2–11: [CuH_6_L]^3+^, [CuH_5_L]^2+^, [CuH_4_L]^+^, [CuH_3_L], [CuH_2_L]^−^, and [CuL]^3–^ and [CuH_–1_L]^4–^, [CuH_–2_L]^5–^, and [CuH_–3_L]^6–^ (Figure 2G). [CuH_6_L]^3+^ and [CuH_5_L]^2+^ most probably arise from the deprotonation of the first and second His residues (maximum concentration at pH 4.0 and 5.0, respectively). Both of the histidine imidazole groups are involved in Cu(II). This is confirmed by the arise of a band at around 700 nm in the UV–vis spectrum at pH 4.01 (corresponding to a bond of Cu(II) with one nitrogen atom) and a shift of this band to 630 nm when the pH increases to 5.21 (corresponding to a bond of Cu(II) with two nitrogen atoms) (Figure S11A).^21,26^ Additionally, this finding is supported by the EPR parameters: *A* = 161.3, *g*_∥_ = 2.31 at pH 4.12 and *A* = 167.2, *g*_∥_ = 2.28 at pH 5.08 (Figure S11B).^21^ The coordination modes for [CuH_6_L]^3+^ and [CuH_5_L]^2+^ are {1N_im_} and {2N_im_}, respectively. The next two species [CuH_4_L]^+^ and [CuH_3_L] (maximum concentration at pH 5.5 and 6.5, respectively) are most likely related to the deprotonation of two non-metal-binding His residues. The p*K*_a_ values of 5.10 and 5.83 for these steps could correspond to p*K*_a_ = 5.79 and 6.29 for two of the His residues in the free ligand, respectively. They are not significantly reduced, suggesting that these residues do not bind Cu(II). The coordination mode for [CuH_4_L]^+^ and [CuH_3_L] is still {2N_im_}. However, in the UV–vis spectrum at pH 6.14–7.11, we can observe the blue shift (630–600 nm) (Figure S11A), suggesting the presence of an equilibrium between species in which two and three nitrogen atoms are involved in Cu(II) binding.^21,26^ This finding is supported by the EPR parameters (*A* = 172.1, *g*_∥_ = 2.26 at pH 6.07; *A* = 182.0, *g*_∥_ = 2.25 at pH 7.10) (Figure S11B)^21,22,33,34^ and is in agreement with potentiometric titrations—at the pH range of 5.5–8.5, another species occurs, [CuH_2_L]^−^ (maximum concentration at pH 7.5). Most probably, it comes from the deprotonation of an imidazole group of the fifth, metal-binding His residue. The p*K*_a_ value of 6.60 for this step could correspond to p*K*_a_ = 7.84 for one of the His residues in the free ligand and is significantly reduced. The coordination mode for [CuH_2_L]^−^ is therefore {3N_im_}. [CuL]^3–^ (maximum concentration at pH 8.5) most probably is related to the deprotonation of one Lys residue and one amide bond. This finding is supported by the arise of a d–d band at 540 and 620 nm in the CD spectrum at pH 8.12, commonly found in Cu(II)–N^–^-type complexes (Figure S11C).^28−31^ Interestingly, this band is present at lower pH compared to CD spectra of Cu(II)-L of previous mutants (Figures S7C–S9C). This is however in agreement with the potentiometric data: in Cu(II)-L systems of L4-L6, [CuL]^3–^ species is less abundant than its co-species—in the case of Cu(II)-L7, [CuL]^3–^ species is slightly more abundant than its co-partner [CuH_2_L]^−^. Additionally, in the UV–vis spectrum at pH 8.12, the presence of a blue shift is observed (600–560 nm), suggesting that there are two species in which three and four nitrogen atoms are present in the Cu(II) coordination sphere (Figure S11A).^21,26^ This is supported by the EPR parameters at pH 8.03: *A* = 192.1, *g*_∥_ = 2.22 (Figure S11B).^21,22,31,32^ The increase of pH to 9.20 (in which [CuL]^3–^ dominates) causes a blue shift (560 to 540 nm), which strongly supports the assumption that four nitrogen atoms bind Cu(II). The coordination mode for [CuL]^3–^ is {3N_im_, 1N^–^}. [CuH_–1_L]^4–^ and [CuH_–2_L]^5–^ (maximum concentration at pH 10.0 and 10.5, respectively) most probably come from the deprotonation of the second and third amide bonds. In the CD spectra at pH 10.03 and 11.02, we can observe that the intensity of the d–d band elevates with increasing pH, suggesting that the formation of a square planar complex and a second and third Cu(II)–N^–^ bond is achieved (Figure S11C).^28−31^ Additionally, in the UV–vis spectrum, we can observe a blue shift (540–525 nm), confirming that at pH 11.02, only 4N-type complex species occur (Figure S11A).^21,26^ The last species, [CuH_–3_L]^6–^, most likely arises from the deprotonation of a second Lys residue. The coordination mode for [CuH_–1_L]^4–^, [CuH_–2_L]^5–^, and [CuH_–3_L]^6–^ is as follows: {2N_im_, 2N^–^}, {1N_im_, 3N^–^}, and {1N_im_, 3N^–^}.

#### Cu(II)-L8

The potentiometric titrations of the Cu(II)-L8 system revealed that eight complex forms exist at the pH range of 2–11: [CuH_6_L]^3+^, [CuH_5_L]^2+^, [CuH_4_L]^+^, [CuH_3_L], [CuHL]^2–^, [CuL]^3–^, and [CuH_-1_L]^4–^ and [CuH_–3_L]^6–^ (Figure 2G). [CuH_6_L]^3+^ and [CuH_5_L]^2+^ most probably come from the deprotonation of the first and second His imidazole groups (maximum concentration at pH 4.5 and 5.0, respectively). Both of the residues are involved in Cu(II) binding. This assumption is supported by the arise of a band at around 700 nm in the UV–vis spectrum at pH 4.11—it is related to the formation of a bond between Cu(II) and one nitrogen atom—and a shift of this band to 640 nm when the pH increases to 5.18 (Figure S12A).^21,26^ This phenomenon is supported by the EPR parameters: *A* = 161.0, *g*_∥_ = 2.31 at pH 4.12 and *A* = 169.5, *g*_∥_ = 2.28 at pH 5.08 (Figure S12B).^21^ The coordination modes for [CuH_6_L]^3+^ and [CuH_5_L]^2+^ should be therefore {1N_im_} and {2N_im_}, respectively. The next species [CuH_4_L]^+^ (maximum concentration at pH 5.5) most likely arises from the deprotonation of the third His imidazole ring. The p*K*_a_ value of 5.10 for this step could be related to p*K*_a_ = 5.80 for one of the His residues in the free ligand. It is only slightly reduced, suggesting that this residue is not involved in Cu(II) binding. In the UV–vis spectrum at pH 6.10–7.12, we can observe a blue shift (630 to 620 and then 610 nm) (Figure S10A), suggesting the presence of an equilibrium of species in which two and three nitrogen atoms are involved in Cu(II) binding.^21,26^ This conclusion is supported by the EPR parameters (*A* = 178.1, *g*_∥_ = 2.26 at pH 6.10; *A* = 179.7, *g*_∥_ = 2.25 at pH 7.12) (Figure S10B).^21,22,34^ It is also in agreement with the potentiometric data—at the pH range of 5.0–8.0, another species occurs, [CuH_3_L] (maximum concentration at pH 6.5) (Figure 2H). Most probably, it comes from the deprotonation of an imidazole group of the fourth His residue. The p*K*_a_ value of 5.68 for this step could correspond to p*K*_a_ = 8.09 for one of the His residues in the free ligand and is significantly reduced—which means that this residue is involved in Cu(II) binding. Coordination modes for [CuH_4_L]^+^ and [CuH_3_L] are {2N_im_} and {3N_im_}, respectively. The next species [CuHL]^2–^ with a maximum concentration at pH 8.0 is most probably related to the deprotonation of one non-metal binding His residue and one amide bond. This is confirmed by the presence of a d–d band in the CD spectrum at pH 8.12, corresponding to the formation of the Cu(II)–N^–^ bond and the change of geometry to square planar (Figure S10C).^28−31^ It suggests that the formation of a complex in which four nitrogen atoms bind Cu(II) starts. In addition, in the UV–vis spectrum at pH 8.12, a slight blue shift was detected (610–585 nm, Figure S10A) and the EPR parameters changed significantly (*A* = 195.3, *g*_∥_ = 2.21, Figure S10B), suggesting that the abundance of a form containing more Cu(II)–N-type bonds increased.^22,32^ The coordination mode for [CuHL]^2–^ is {3N_im_, 1N^–^}.^21^ Two other species [CuL]^3–^ and [CuH_-1_L]^4–^ (maximum concentration at pH 9.0 and 10.0, respectively) most likely arise from the deprotonation of another two amide bonds. We can observe two effects that confirm this result: (1) the presence of a blue shift at pH 9.01 (585–560 nm) and then at pH 10.10 (560–540 nm) suggests the increase of the species in which four nitrogen atoms bind Cu(II) (Figure S12A) and (2) the d–d band in the CD spectrum becomes more abundant (Figure S12C). An increase of the pH to 11.06 does not significantly impact the parameters of CD and UV–vis spectra. This is in agreement with the potentiometric data—the most abundant species at pH around 11.0 is [CuH_–3_L]^6–^ that presumably is related to the deprotonation of two non-metal-binding Lys residue.

#### Secondary Structure Studies

Under the conditions of the CD experiment at pH 2–11 that were adjusted to potentiometric titrations, all of the studied ligands exhibit a random-coil structure. An example of the CD spectrum of ligand L2 is presented in Figure S13A. The presence of Cu(II) does not significantly impact this correlation (Figure S13B). Peptides are not likely to form secondary and tertiary structures, compared to native proteins. Some amino acid residues, such as Gly/Pro (common “helix breaker”) or with amphiphilic side chains having both, a polar group and a flexible hydrocarbon such as His, Lys, Glu, Arg, and Gln additionally decrease the chances of peptides to form secondary structures.^35^ This is in agreement with the results of XRD study of GroEL1 (*M. tuberculosis*)—the solved structure of this protein lacks approximately 20 amino acids on the C-terminus, due to the high flexibility of this fragment.^13^

## Discussion

### Stability of Cu(II) Complexes

**Figure 3:**
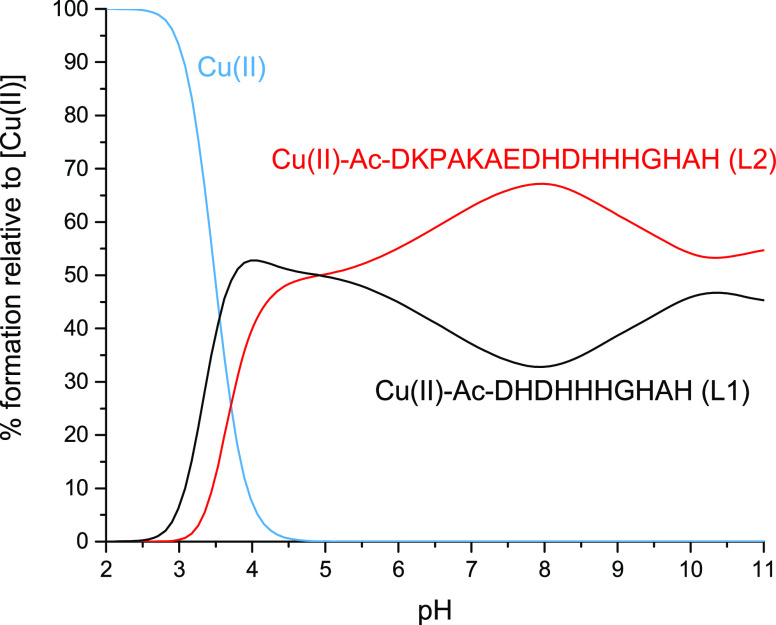
Competition plot between L1 and L2 ligand complexes with the Cu(II) ion, showing complex formation in a hypothetical solution in which the metal ion and two peptides are present. The calculation based on the potentiometric data for the studied systems is given in the table.

**Figure 4:**
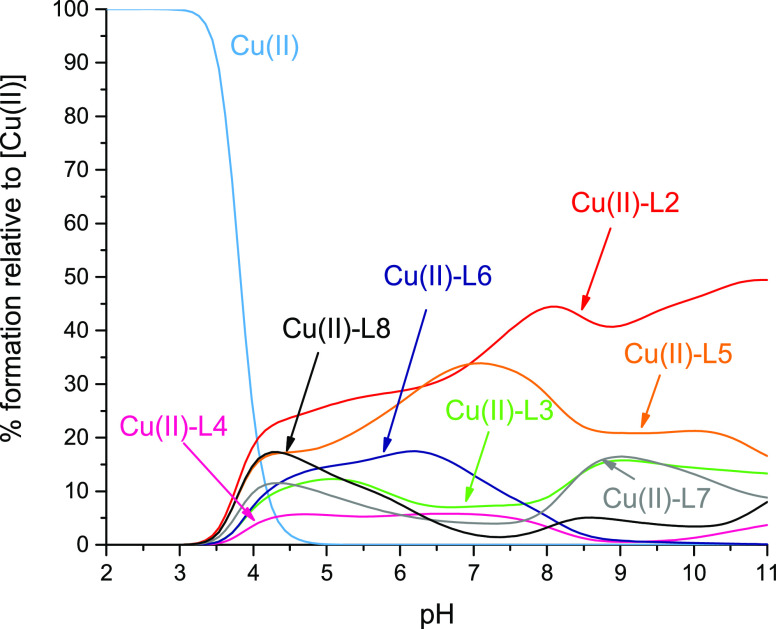
Competition plot between L2–L8 complexes with the Cu(II) ion, showing complex formation in a hypothetical solution in which the metal ion and seven peptides are present. The calculation based on the potentiometric data for the studied systems is given in the table.

To compare the stability of the studied copper complexes and the affinity of different ligands for the Cu(II) ion, the competition plots were drawn (Figures 3 and 4). These graphs are based on the protonation and stability constants and show a hypothetical situation in which equimolar concentrations of all reagents are present. They are theoretical, based on the potentiometric data. Figure 3 reveals that the longer counterpart (L2) forms a more stable complex with Cu(II) than the shorter one (L1). This can be easily explained: the potentiometric and spectroscopic studies (Figures S5 and S6) revealed that in L1, two His residues are most likely to bind Cu(II) with three His residues in the case of L2. Theoretical explanation can also be given: L2 is more hydrophilic, so in aqueous solutions, it will show better properties as a ligand because of its significant amount of charged amino acid residues (e.g., Lys and Asp). These residues should stabilize the complex through the formation of ion pairs, hydrogen bonds, and other less specific electrostatic interactions.^36,37^ The formation of Lys–Asp interactions, so called “salt bridges”, is possible. Nevertheless, we do not observe the α-helix formation (Figure S13A,B), perhaps due to the conditions of the CD experiments and the presence of the Pro residue and many His residues.^35^ Another explanation can be given by discussing the charge of the peptides: in the initial phase, i.e., low pH, acidic residues deprotonate. Then, the positive sum charge of L2 is higher than that of L1; therefore, L1 forms slightly more stable complexes with Cu(II) at the low pH of 3.0–4.0. With the deprotonation of successive His residues (above pH around 4.3), this correlation is reversed and Cu(II)-L2 is more stable than Cu(II)-L1 because it is less positively charged. As a result of the deprotonation of Lys residues in L2, the electrostatic interactions of Asp–Lys significantly weaken, and the stabilities of Cu(II)-L2 and Cu(II)-L1 at pH above 9.5 are again very similar.

Which His mutations significantly impact the stability of the Cu(II) complex? The answer to this question is intriguing. It is clear that even in the absence of one His residue, the ligand is still able to bind Cu(II). We can see it from the results of the potentiometric, MS, and spectroscopy experiments (Figures 2 and S1–S14). However, we can also observe that the original L2 is more likely to interact with the Cu(II) ion: in the previous sections, we discussed that L2 reaches the {3N_im_} coordination mode at pH 6.07 (Figure S6). All of the mutations “disrupt” the system: we can see that all of the mutants can finally bind three His residues (Figures S7–S12), but these systems struggle to find this third binding site for the metal ion: in the 6.0–8.0 pH range, we can observe an equilibrium of species in which two or three nitrogen atoms bind Cu(II). This indicates that the deletion of one His residue weakens the coordination properties of L3–L8 ligands. We can observe this correlation in Figure 4. It also shows that in the most biologically important physiological pH around 7.4, the stability of the Cu(II) complex with all mutants except L5 is very similar but lower compared to that of Cu(II)-L2. The reason for it could be that every positively charged His residue forms strong electrostatic interactions with negatively charged amino acid residues such as Glu and Asp and C-terminus that stabilize the structure of peptides, proteins, and their metal complexes.^38^ On the other hand, even in the presence of five His residues, not six (like in L2), the system manages to finally reach the {3N_im_} coordination mode. What could this mean for the biological role of GroEL1? If an undesirable, accidental mutation occurs and the HRCT will end with less than six potential binding sites, the GroEL1 could still function as a metal transporter. This is very important for the whole biological system. It also indicates one more thing: most probably, there is not only one proper binding mode for GroEL1 HRCT but a few possible binding modes.

**Figure 5:**
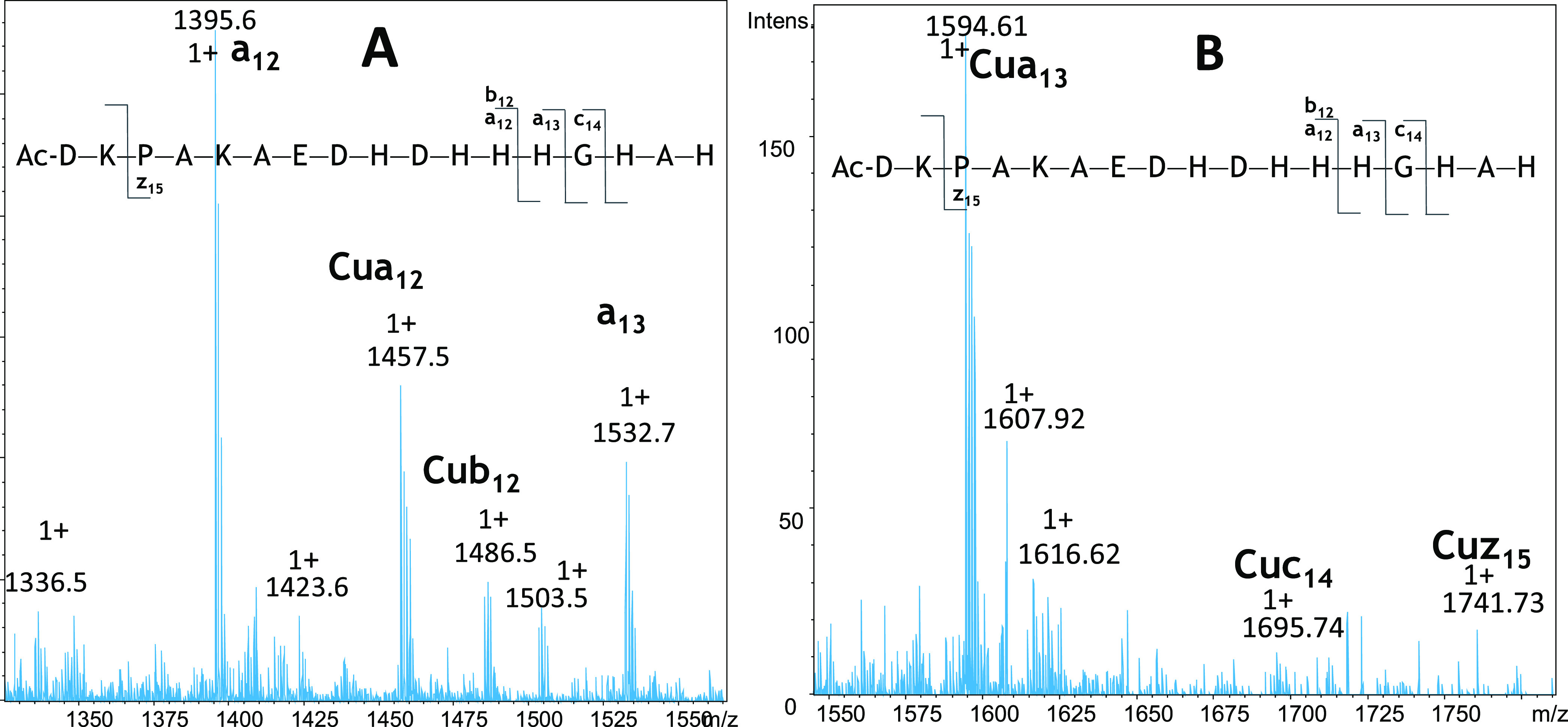
ESI-MS/MS spectrum of the Cu(II)-L2 sample at (A) 1325–1575 *m*/*z* range and (B) 1550–1775 *m*/*z* range. Parent ion *m*/*z* = 1021.93, *z* = 2+; collision energy 40 eV. The fragmentation pattern was assigned.^40^

However, is it possible to point out which “side” of the GroEL1 HRCT has greater affinity for Cu(II)? The most accurate methods for these studies could be NMR spectroscopy or XRD crystallography. In our systems, the Cu(II) paramagnetic properties and the very close neighborhood of each His residue do not make it straightforward to obtain informative NMR spectra. The flexibility and inability to form secondary structures of our ligands—a property mentioned before in the Introduction section and confirmed by CD secondary structure studies—revealed that XRD is also not a suitable analytical method in this case. Therefore, we tried another approach: tandem mass spectrometry studies. We isolated and fragmented the parent ions of [L]^2+^ (*m*/*z* = 991.44, *z* = +1) and [CuL]^2+^ (*m*/*z* = 1021.93, *z* = +2) of L2, and the results of these experiments are presented in Figures S14 and 5. The comparison of the two spectra revealed in Figure 5 additional signals corresponding to ions of Cu(II)-containing fragments: Cua_12_, Cua_13_, Cub_12_, Cuc_14_, and Cuz_15_—they correspond to a Cu(II) complex of appropriate fragments. The presence of the above-mentioned signals in the MS/MS spectrum strongly suggests that three of the His9–His13 residues, Ac-DKPAKAED**H**D**HHH**, preferably bind Cu(II). This is in agreement with the potentiometric results—the L4 mutant lacking the His11 residue forms the least stable complex with Cu(II) in the widest pH range. The reason for this preference might be that the close neighborhood of two Asp residues in this region additionally supports Cu(II) binding—the Asp residue itself is known for being a binding site for Cu(II), although Cu(II) prefers the His residue.^39^ If His11 was the main binding site for Cu(II) in L2–L3 and L5–L8, the three amide bonds involved in metal binding at high pH above 9 would preferably belong to the two next amino acid residues on the left: Ac-DKPAKAEDHDHHHGHAH (in the N-terminus direction)—the presence of negatively charged Asp residues could stabilize the metal ion on this site.

Another interesting result is that Cu(II)-L5 has a very similar stability as that of Cu(II)-L2 in the wide pH range. What can be the reason for it? The sequence of Ac-DKPAKAED**H**D**H**Q**H**G**H**A**H** (L5) has a very special pattern: all His residues are equally remote from each other, by one amino acid residue. It suggests that the Cu(II) ion has a similar distance to all of its potential binding sites, and this simple regularity could be a reason for additional stability to the complex system.^47^ GroEL1 proteins of different *Mycobacteria* species possess a repeatable pattern of His residue arrangement at the C-terminus: -DKPAEEADD**H**G**H**G**H**H**H**H (*Mycobacterium avium* (strain 104), UniProt code: CH601_MYCA1),^41^ -EKPAEAEDDG**H**G**H**G**H**G**H**H**H**H (*Mycolicibacterium gilvum* (strain PYR-GCK), UniProt code: CH601_MYCGI),^42^ -DEPANAHE**H**D**H**G**H**G**H**G**H**H**H**H**H** (*Mycobacterium kansasii*, UniProt code: A0A653F4Z6_MYCKA),^43^ -DKPAKAED**H**D**H**H**H**G**H**A**H** (*Mycobacterium bovis* (*strain ATCC BAA-935/AF2122/97*), UniProt code: A0A679LKC3_MYCBO),^44^ and -DKPAEEADD**H**G**H**G**H**H**H**H (*Mycolicibacterium paratuberculosis(strain ATCC BAA-968/K-10*) *(Mycobacterium paratuberculosis)*, UniProt code: CH601_MYCPA).^45^ It supports the suspicion that the probable binding sites are arranged similarly like in the case of L5—at the same distance from each other. This would be in agreement with our findings and could support the explanation of the competition plot (Figure 4).

We do not exclude the possibility to form binuclear complexes in the presence of an excess of metal ions. MS studies at pH 7.5 revealed that all L2–L8 ligands tend to form stable binuclear complexes with Cu(II), when the mixture of M/L = 2:1 is applied (Figures S2B, S3B, S4A–E). However, we can see that in the case of particular mutants, this tendency along with the ability to be ionized decreases or increases, compared to the original sequence. It seems like the mutation on His11 significantly disrupts the stability of Cu_2_L species under the ESI conditions or relatively prevents from binding the second Cu(II) ion. Mutation on His9 and His12 causes the increase of Cu_2_L species abundance (Figures S3B and S4B). Interestingly, His13, His15, and His17 mutations cause the huge increase of Cu_2_L signal intensity (Figure S4C–E). Opposite to the other mutations, these also caused the significant decrease of mononuclear species. We could assume that the His11 is the most important residue for the binuclear species stability in the presence of Cu(II) ion excess and is an important Cu(II)-binding site, which is in agreement with the MS/MS result and also supports the competition plot of the equimolar M/L mixture (Figures S4A, 4, and 5). On the other hand, His13, His15, and His17 residues seem to prevent the binuclear species formation under the ESI experiment conditions and may not be so important in Cu(II) binding. His11–12 most probably are also important for Cu_2_L species stability and could provide binding sites for Cu(II).

## Conclusions

In this work, we fully characterized the properties of the mycobacterial GroEL1 C-terminus and its mutants. We established that in the equimolar mixture of reagents, all of the ligands form equimolar complexes with Cu(II). In the case of L2−L8, the coordination sphere of the metal ion most probably is occupied by three His residues, and in the case of L1, it is occupied by two His residues. The amino acid residues adjacent to the HRCT fragment significantly increase the stability of the complexes. The mutations on all six His residues do not impact the number of the binding sites for Cu(II) but affect the stability of the complexes—it is lower for the peptides containing five His residues instead of six. Surprisingly, L5 appeared to be the most stable of all mutants—this is probably due to its unique His arrangement, equally remote from each other, by one amino acid residue. This could increase the chances of reaching the Cu(II) coordination sphere equally for all His residues of L5 and elevate the stability of this system. Most probably, in the case of L2, the metal-binding sites are His residues arranged closer to the “left side” of the peptides—the stability of Cu(II)-L4, in which the His11 residue is replaced with the Gln residue, is the lowest in the widest pH range. This is supported by the MS/MS fragmentation spectral analysis.

## Supporting Information Available

Complementary mass spectra, UV–vis/CD/EPR spectra, and MS/MS spectrum (PDF)
