# Supplementary material for: Histidine-Rich C-Terminal Tail of Mycobacterial GroEL1 and Its Copper Complex—The Impact of Point Mutations
Source: Inorg Chem. 2023 Apr 24;62(18):6893–908. doi: 10.1021/acs.inorgchem.2c04486 (PMC10170517; doi:10.1021/acs.inorgchem.2c04486)
Supplement: Supplementary file 1 — ic2c04486_si_001.pdf [file ic2c04486_si_001.pdf]

Histidine-Rich C-Terminal tail of Mycobacterial GroEL1 and Its Copper Complex – The Impact of Point Mutations

Anna Rola[1*], Oscar Palacios[2], Merce Capdevila[2], Daniela Valensin[3], Elżbieta Gumienna-Kontecka[1], Sławomir Potocki[1*]

slawomir.potocki@chem.uni.wroc.pl

[1]Faculty of Chemistry, University of Wroclaw, 50- 383 Wroclaw, Poland;

[2]Departament de Química, Universitat Autònoma de Barcelona, 08193 Cerdanyola del Vallès, Spain;

[3] Department of Biotechnology, Chemistry and Pharmacy, University of Siena, Via A. Moro 2, 53100 Siena, Italy

Table S1. The major coordination modes of Cu(II)-L system at pH range 4.0-11.0, where L means the appropriate model peptide. The abundance of the selected species is above 10%. Most dominant form at precise pH is bold.

	Major species and proposed coordination mode							
**pH**	**L1**	**L2**	**L3**	**L4**	**L5**	**L6**	**L7**	**L8**
**4.0**	**CuH$_5$L** {1N$_{im}$}	**CuH$_8$L** {1N$_{im}$}	**CuH$_6$L** {1N$_{im}$}	**CuH$_6$L** {1N$_{im}$}	**CuH$_6$L** {1N$_{im}$}	**CuH$_6$L** {1N$_{im}$}	**CuH$_6$L** {1N$_{im}$}	**CuH$_6$L** {1N$_{im}$}
	**CuH$_4$L** {2N$_{im}$}	**CuH$_7$L** {1N$_{im}$}			CuH$_5$L {2N$_{im}$}	CuH$_5$L {2N$_{im}$}	CuH$_5$L {2N$_{im}$}	
		CuH$_6$L {2N$_{im}$}						
**5.0**	CuH$_4$L {2N$_{im}$}	CuH$_6$L {2N$_{im}$}	CuH$_6$L {1N$_{im}$}	CuH$_6$L {1N$_{im}$}	CuH$_5$L {2N$_{im}$}	CuH$_6$L {1N$_{im}$}	CuH$_6$L {1N$_{im}$}	CuH$_6$L {1N$_{im}$}
	**CuH$_3$L** {2N$_{im}$}	**CuH$_5$L** {2N$_{im}$}	CuH$_5$L {2N$_{im}$}	**CuH$_5$L** {2N$_{im}$}	**CuH$_4$L** {2N$_{im}$}	**CuH$_5$L** {2N$_{im}$}	**CuH$_5$L** {2N$_{im}$}	**CuH$_5$L** {2N$_{im}$}
	CuH$_2$L {2N$_{im}$}	CuH$_4$L {3N$_{im}$}	**CuH$_4$L** {2N$_{im}$}	CuH$_4$L {2N$_{im}$}	CuH$_3$L {2N$_{im}$}	CuH$_4$L {2N$_{im}$}	CuH$_4$L {2N$_{im}$}	CuH$_4$L {2N$_{im}$}
**6.0**	CuH$_3$L {2N$_{im}$}	**CuH$_4$L** {3N$_{im}$}	**CuH$_4$L** {2N$_{im}$}	CuH$_4$L {2N$_{im}$}	CuH$_4$L {2N$_{im}$}	CuH$_4$L {2N$_{im}$}	CuH$_4$L {2N$_{im}$}	CuH$_4$L {2N$_{im}$}
	**CuH$_2$L** {2N$_{im}$}	CuH$_3$L {3N$_{im}$}	CuH$_3$L {2N$_{im}$}	**CuH$_3$L** {2N$_{im}$}	**CuH$_3$L** {2N$_{im}$}	**CuH$_3$L** {3N$_{im}$}	**CuH$_3$L** {2N$_{im}$}	**CuH$_3$L** {3N$_{im}$}
	CuHL {2N$_{im}$}		CuH$_2$L {3N$_{im}$}	CuH$_2$L {3N$_{im}$}	CuH$_2$L {3N$_{im}$}		CuH$_2$L {3N$_{im}$}	
**7.0**	CuHL {2N$_{im}$}	CuH$_3$L {3N$_{im}$}	CuH$_3$L {2N$_{im}$}	CuH$_3$L {2N$_{im}$}	CuH$_3$L {2N$_{im}$}	**CuH$_3$L** {3N$_{im}$}	CuH$_3$L {2N$_{im}$}	**CuH$_3$L** {3N$_{im}$}
	**CuL** {2N$_{im}$}	**CuH$_2$L** {3N$_{im}$}	**CuH$_2$L** {3N$_{im}$}	**CuH$_2$L** {3N$_{im}$}	**CuH$_2$L** {3N$_{im}$}	CuH$_2$L {3N$_{im}$}	**CuH$_2$L** {3N$_{im}$}	CuHL {3N$_{im}$. 1N$^-$}
**8.0**	**CuL** {2N$_{im}$}	**CuH$_2$L** {3N$_{im}$}	**CuH$_2$L** {3N$_{im}$}	**CuH$_2$L** {3N$_{im}$}	**CuH$_2$L** {3N$_{im}$}	CuH$_3$L {3N$_{im}$}	CuH$_2$L {3N$_{im}$}	**CuHL** {3N$_{im}$. 1N$^-$}
	CuH$_{-1}$L {2N$_{im}$. 1N$^-$}	CuHL {3N$_{im}$}	**CuL** {3N$_{im}$. 1N$^-$}		CuL {3N$_{im}$. 1N$^-$}	**CuH$_2$L** {3N$_{im}$}	**CuL** {3N$_{im}$. 1N$^-$}	CuL {2N$_{im}$. 2N$^-$}
	CuH$_{-2}$L {2N$_{im}$. 2N$^-$}							
**9.0**	CuH$_{-2}$L {2N$_{im}$. 2N$^-$}	**CuL** {3N$_{im}$}	**CuL** {3N$_{im}$. 1N$^-$}	CuH$_2$L {3N$_{im}$}	**CuL** {3N$_{im}$. 1N$^-$}	CuH$_2$L {3N$_{im}$}	**CuL** {3N$_{im}$. 1N$^-$}	**CuL** {2N$_{im}$. 2N$^-$}
	**CuH$_{-3}$L** {1N$_{im}$. 3N$^-$}	**CuH$_{-1}$L** {3N$_{im}$. 1N$^-$}	CuH$_{-1}$L {2N$_{im}$. 2N$^-$}	**CuL** {2N$_{im}$. 2N$^-$}	CuH$_{-1}$L {2N$_{im}$. 2N$^-$}	**CuL** {3N$_{im}$. 1N$^-$}	CuH$_{-1}$L {2N$_{im}$. 2N$^-$}	CuH$_{-1}$L {1N$_{im}$. 3N$^-$}
				CuH$_{-1}$L {2N$_{im}$. 2N$^-$}		CuH$_{-1}$L {2N$_{im}$. 2N$^-$}		
**10.0**	CuH$_{-3}$L {1N$_{im}$. 3N$^-$}	**CuH$_{-1}$L** {3N$_{im}$. 1N$^-$}	CuL {3N$_{im}$. 1N$^-$}	CuH$_{-1}$L {2N$_{im}$. 2N$^-$}	CuL {3N$_{im}$. 1N$^-$}	CuL {3N$_{im}$. 1N$^-$}	CuL {3N$_{im}$. 1N$^-$}	CuL {2N$_{im}$. 2N$^-$}
		CuH$_{-2}$L {2N$_{im}$. 2N$^-$}	**CuH$_{-1}$L** {2N$_{im}$. 2N$^-$}	**CuH$_{-2}$L** {1N$_{im}$. 3N$^-$}	**CuH$_{-1}$L** {2N$_{im}$. 2N$^-$}	**CuH$_{-1}$L** {2N$_{im}$. 2N$^-$}	**CuH$_{-1}$L** {2N$_{im}$. 2N$^-$}	**CuH$_{-1}$L** {1N$_{im}$. 3N$^-$}
		CuH$_{-3}$L {1N$_{im}$. 3N$^-$}	CuH$_{-2}$L {1N$_{im}$. 3N$^-$}	CuH$_{-3}$L {1N$_{im}$. 3N$^-$}	CuH$_{-2}$L {1N$_{im}$. 3N$^-$}	CuH$_{-2}$L {1N$_{im}$. 3N$^-$}	CuH$_{-2}$L {1N$_{im}$. 3N$^-$}	CuH$_{-3}$L {1N$_{im}$. 3N$^-$}
**11.0**	CuH$_{-3}$L {1N$_{im}$. 3N$^-$}	CuH$_{-2}$L {2N$_{im}$. 2N$^-$}	CuH$_{-2}$L {1N$_{im}$. 3N$^-$}	CuH$_{-2}$L {1N$_{im}$. 3N$^-$}	CuH$_{-1}$L {2N$_{im}$. 2N$^-$}	CuH$_{-1}$L {2N$_{im}$. 2N$^-$}	CuH$_{-1}$L {2N$_{im}$. 2N$^-$}	CuH$_{-1}$L {1N$_{im}$. 3N$^-$}
		**CuH$_{-3}$L** {1N$_{im}$. 3N$^-$}	**CuH$_{-3}$L** {1N$_{im}$. 3N$^-$}	**CuH$_{-3}$L** {1N$_{im}$. 3N$^-$}	**CuH$_{-2}$L** {1N$_{im}$. 3N$^-$}	**CuH$_{-2}$L** {1N$_{im}$. 3N$^-$}	CuH$_{-2}$L {1N$_{im}$. 3N$^-$}	**CuH$_{-3}$L** {1N$_{im}$. 3N$^-$}
					CuH$_{-3}$L {1N$_{im}$. 3N$^-$}	CuH$_{-3}$L {1N$_{im}$. 3N$^-$}	**CuH$_{-3}$L** {1N$_{im}$. 3N$^-$}	

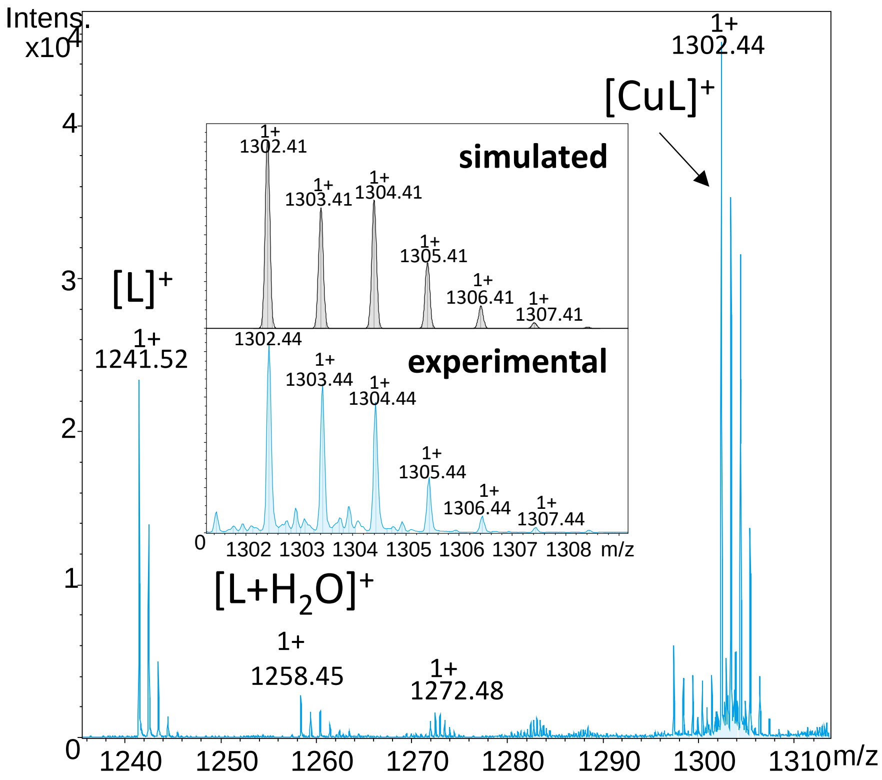

Figure S1. ESI-MS spectrum of a Cu(II)-Ac-DHDHHHGHAH (L1) system in the m/z 1235–1315 range at pH 7.5 [M/L = 1:1]. The simulated and experimental isotopic distribution spectra of the peak at *m/z* = 1302.44 are presented in the middle.

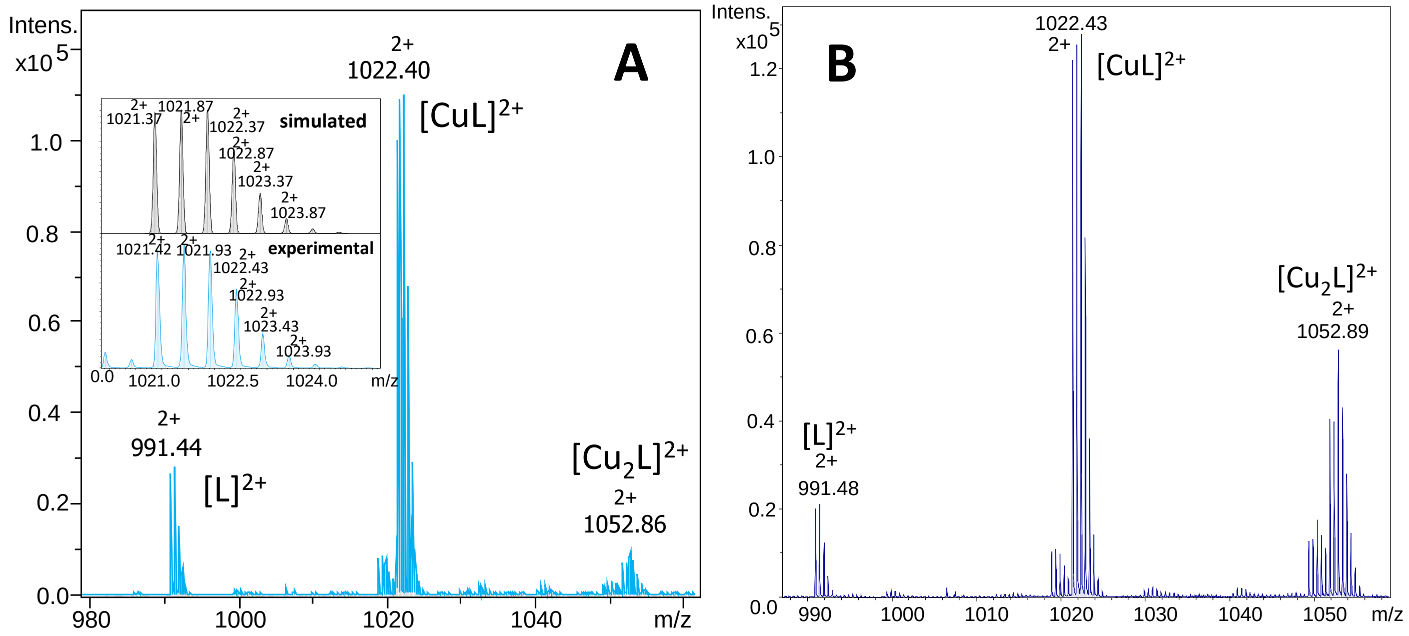

Figure S2. ESI-MS spectrum of a Cu(II)-Ac-DKPAKAEDHDHHHGHAH (L2) system in the m/z 980–1060 range at pH 7.5 (A), [M/L = 1:1]; (B) [M/L = 2:1].The simulated and experimental isotopic distribution spectra of the peak at *m/z* = 1022.40 are presented in the middle of (A).

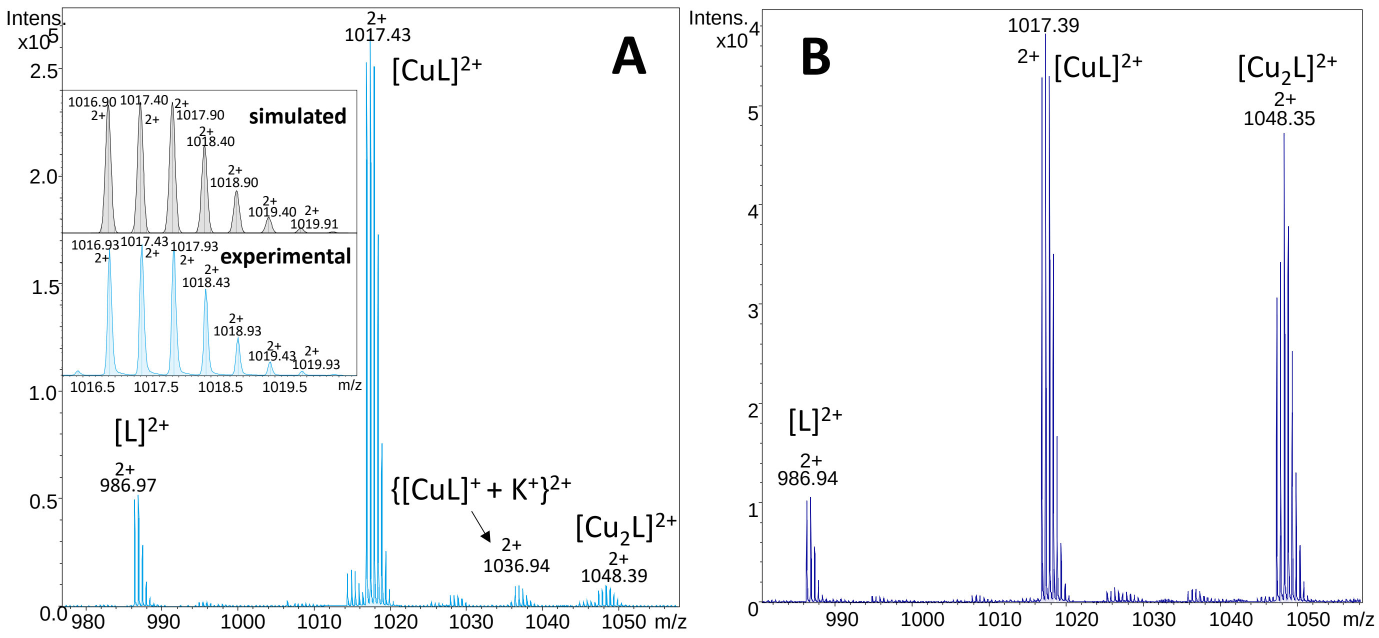

Figure S3. ESI-MS spectrum of a Cu(II)-Ac-DKPAKAEDQDHHHGHAH (L3) system in the m/z 970–1060 range at pH 7.5 (A), [M/L = 1:1]; (B) [M/L = 2:1].The simulated and experimental isotopic distribution spectra of the peak at *m/z* = 1017.43 are presented in the middle of (A). The spectrum is identical in case of all studied mutants.

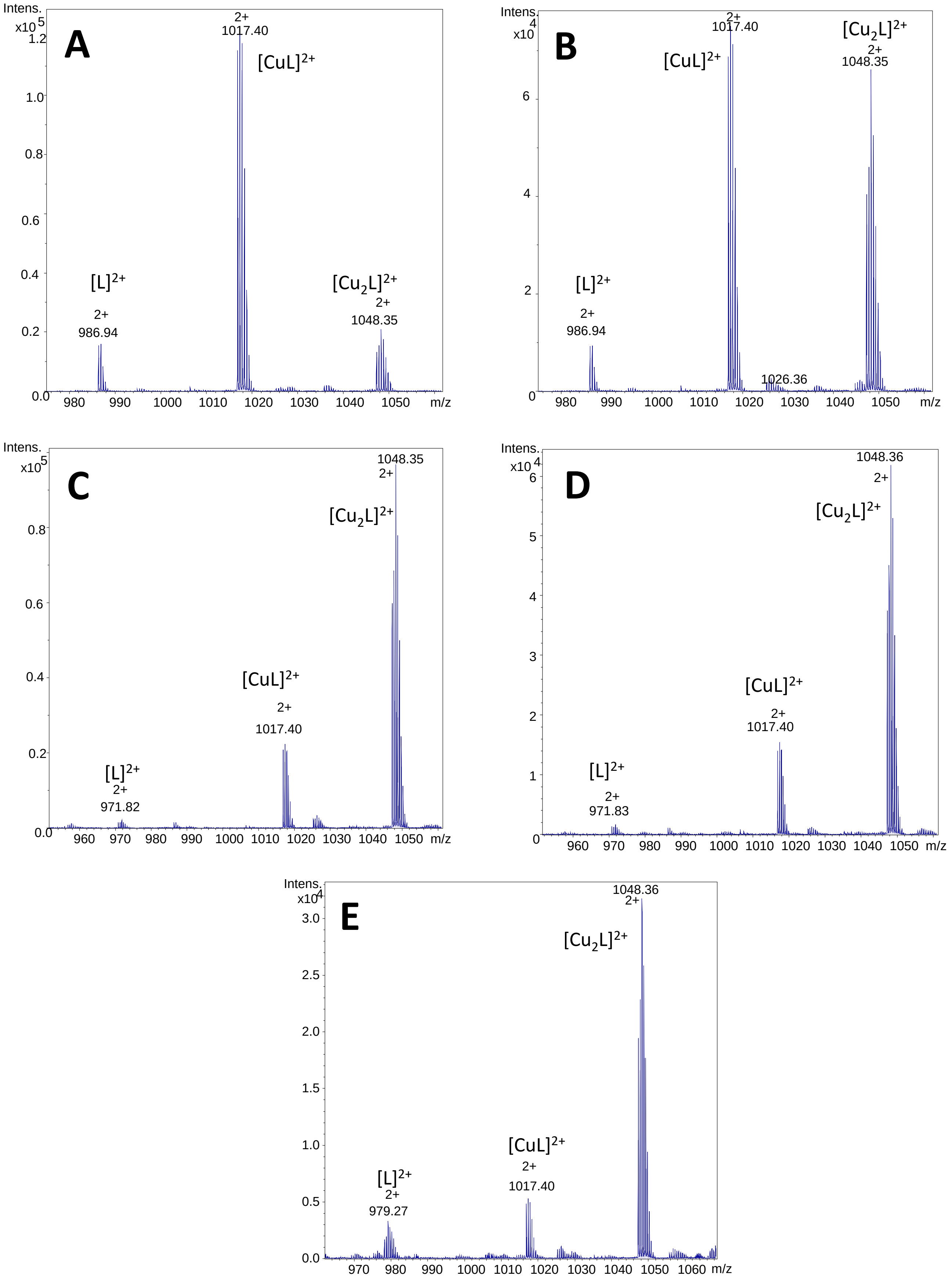

Figure S4. ESI-MS spectrum of a (A) Cu(II)-Ac-DKPAKAEDHD**Q**HHGHAH (L4); (B) Cu(II)-Ac-DKPAKAEDHDH**Q**HGHAH (L5); (C) Cu(II)-Ac-DKPAKAEDHDHH**Q**GHAH (L6); (D) Cu(II)-Ac-DKPAKAEDHDHHHG**Q**AH (L7); (E) Cu(II)-Ac-DKPAKAEDHDHHHGHA**Q** (L8) system in the m/z 970–1060 range at pH 7.5 [M/L = 2:1].

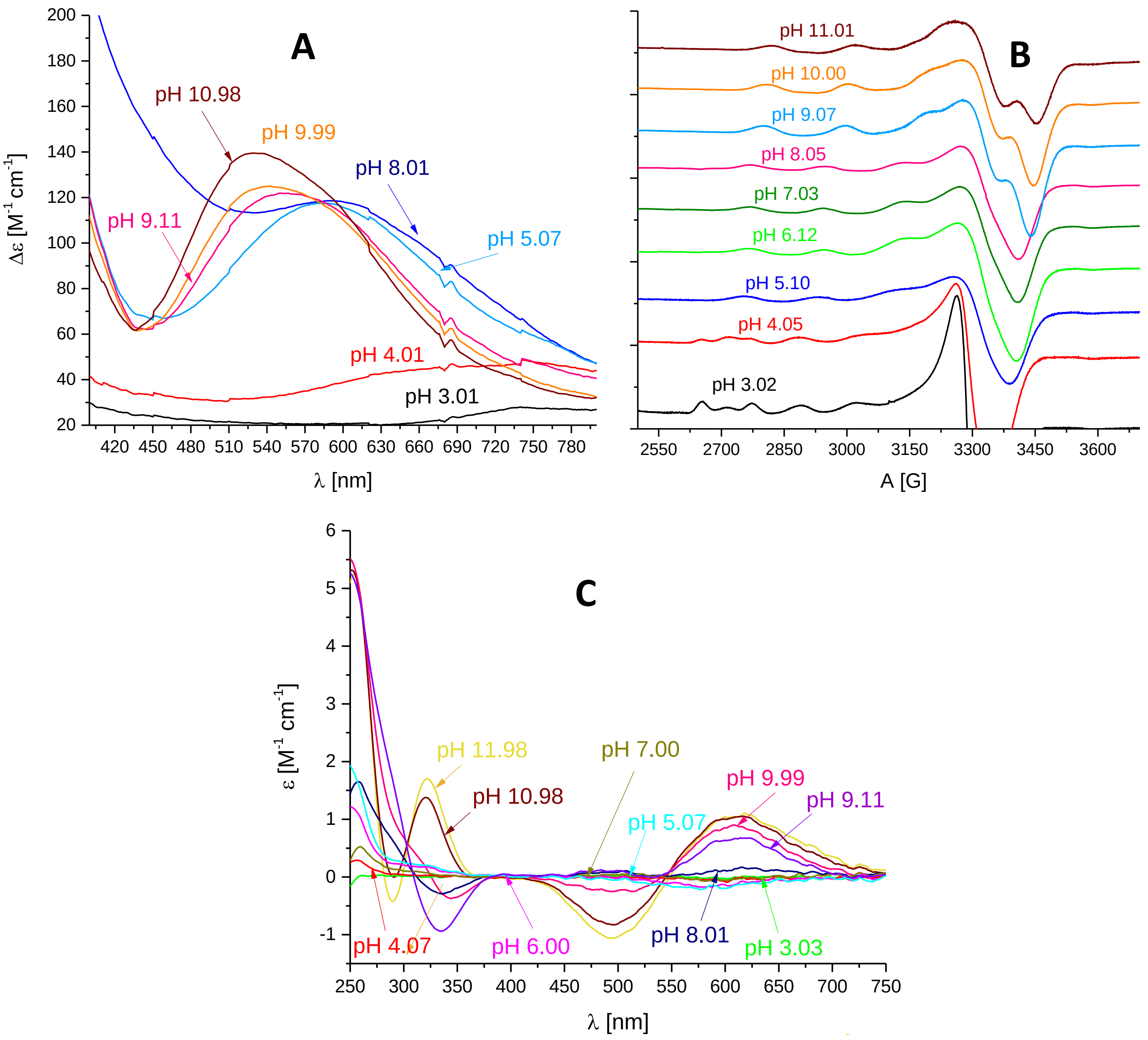

Figure S5. UV-Vis (A), EPR (B) and CD (D) spectra of Cu(II)-L1 system over the pH range 2–11. Conditions: T = 298 K and metal to ligand ratio = 0.8:1; [Cu(II)] = 4 × 10^{-4} M.

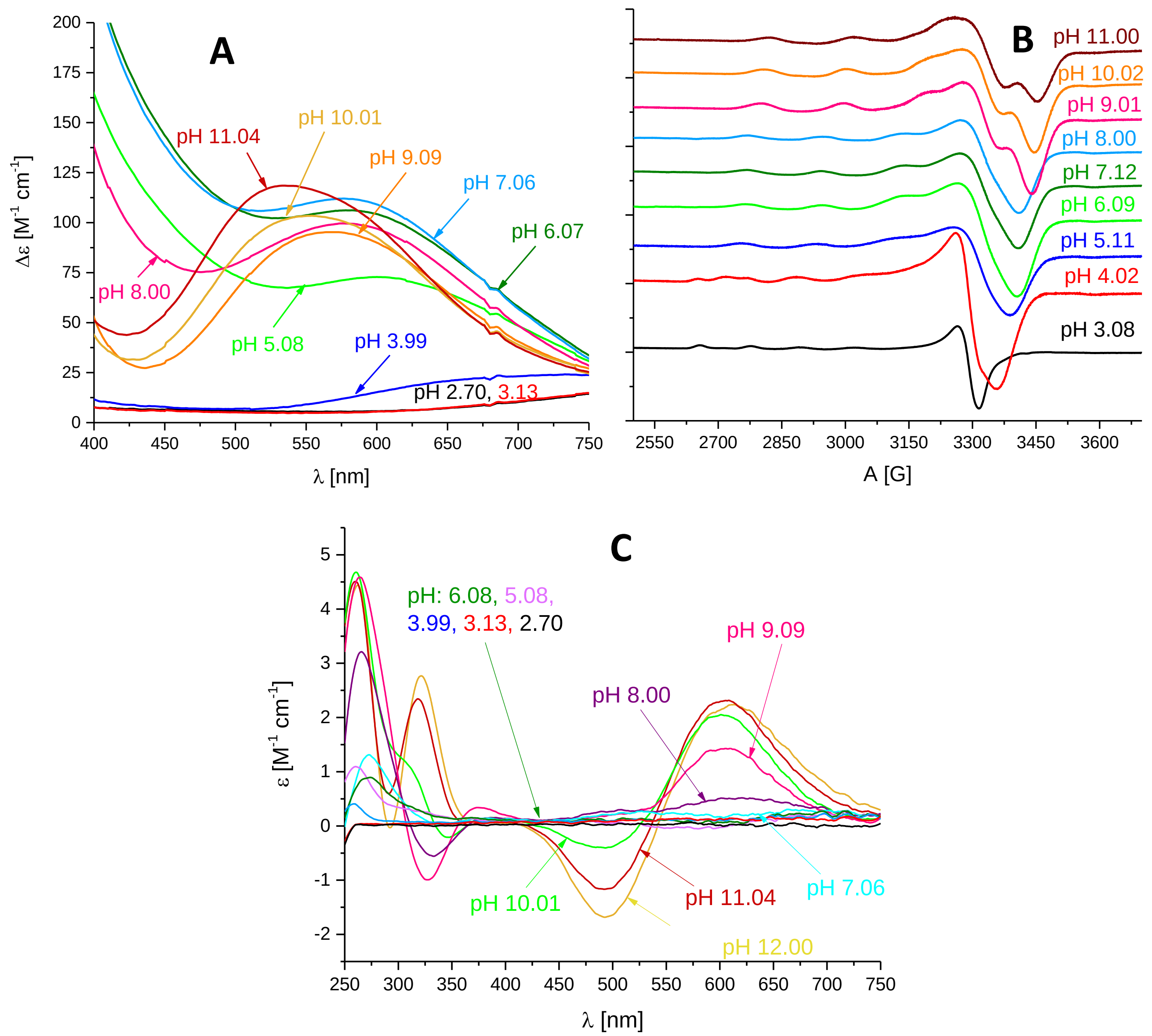

Figure S6. UV-Vis (A), EPR (B) and CD (D) spectra of Cu(II)-L2 system over the pH range 2–11. Conditions: T = 298 K and metal to ligand ratio = 0.8:1; [Cu(II)] = 4 × 10^{-4} M.

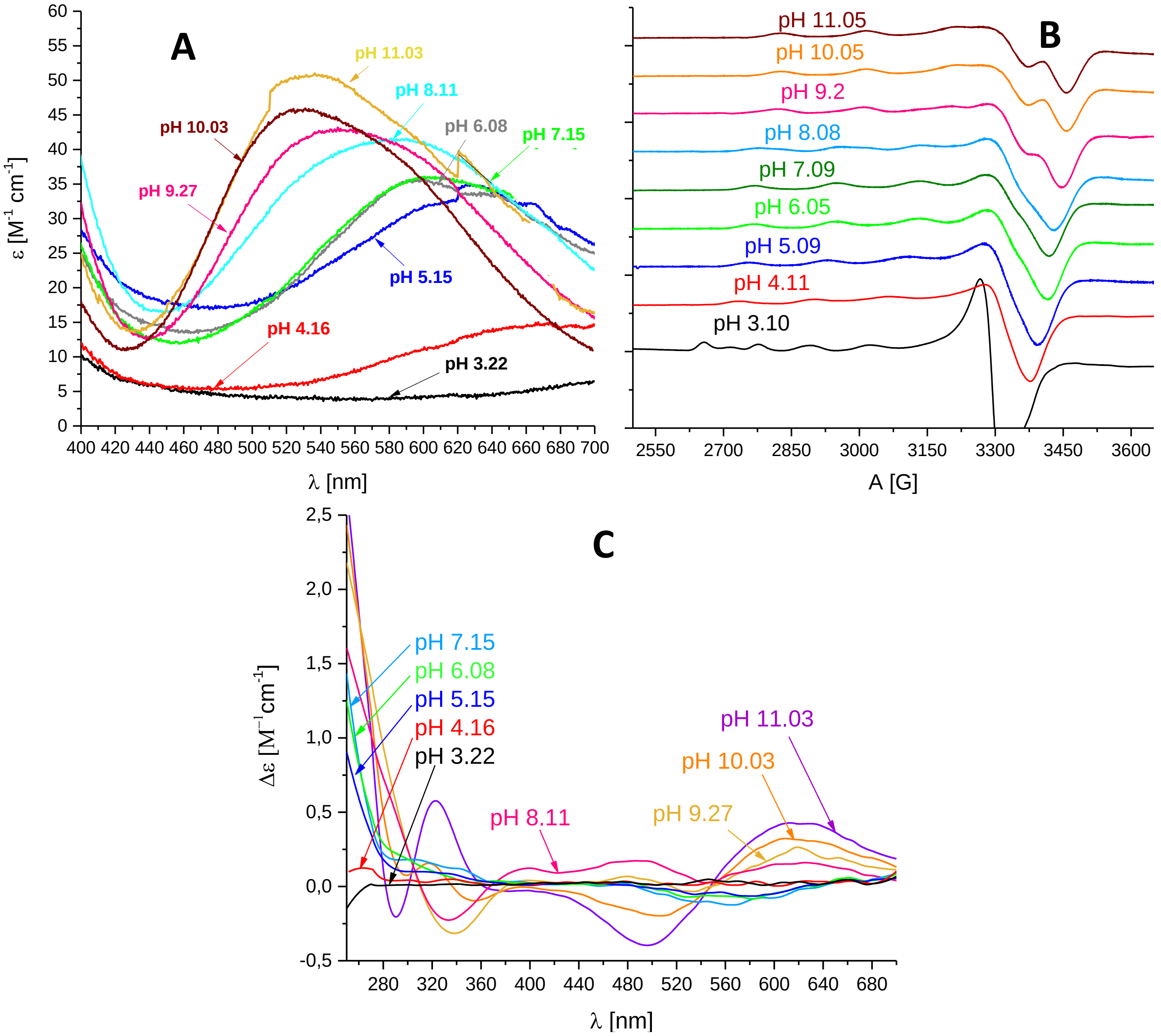

Figure S7. UV-Vis (A), EPR (B) and CD (D) spectra of Cu(II)-L3 system over the pH range 2–11. Conditions: T = 298 K and metal to ligand ratio = 0.8:1; [Cu(II)] = 4 × 10^{-4} M.

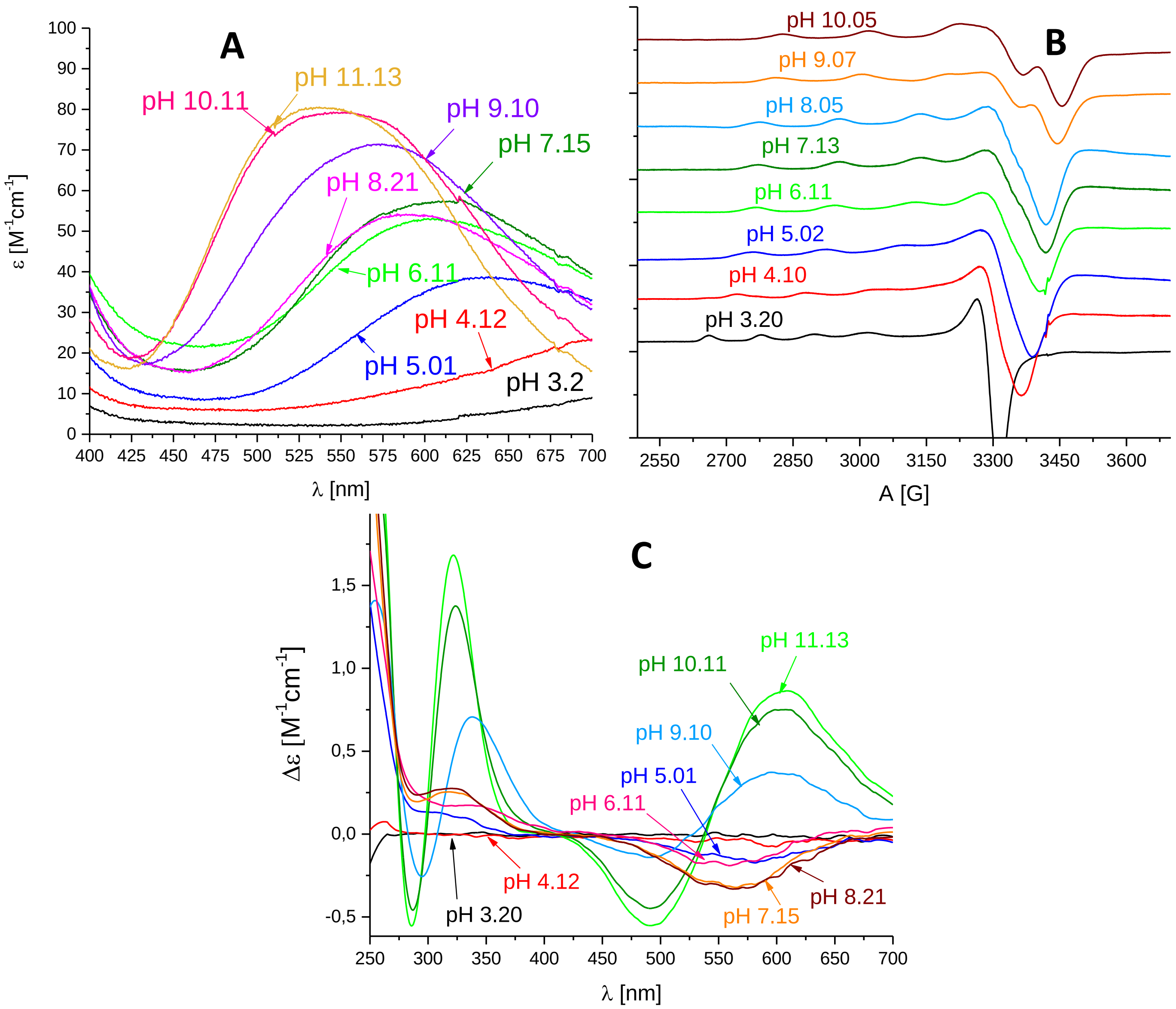

Figure S8. UV-Vis (A), EPR (B) and CD (D) spectra of Cu(II)-L4 system over the pH range 2–11. Conditions: T = 298 K and metal to ligand ratio = 0.8:1; [Cu(II)] = 4×10^{-4} M.

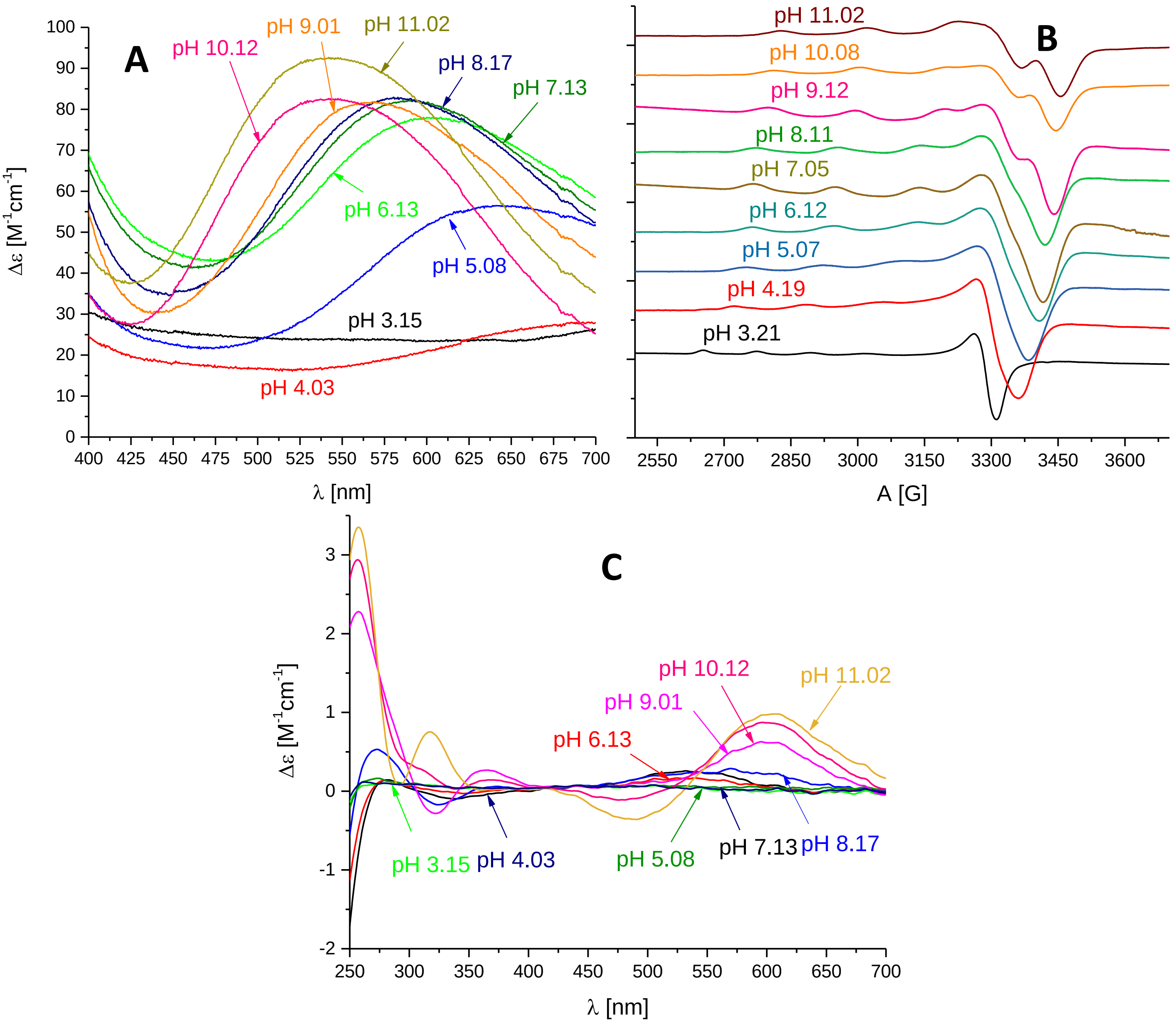

Figure S9. UV-Vis (A), EPR (B) and CD (D) spectra of Cu(II)-L5 system over the pH range 2–11. Conditions: T = 298 K and metal to ligand ratio = 0.8:1; [Cu(II)] = 4 × 10^{-4} M.

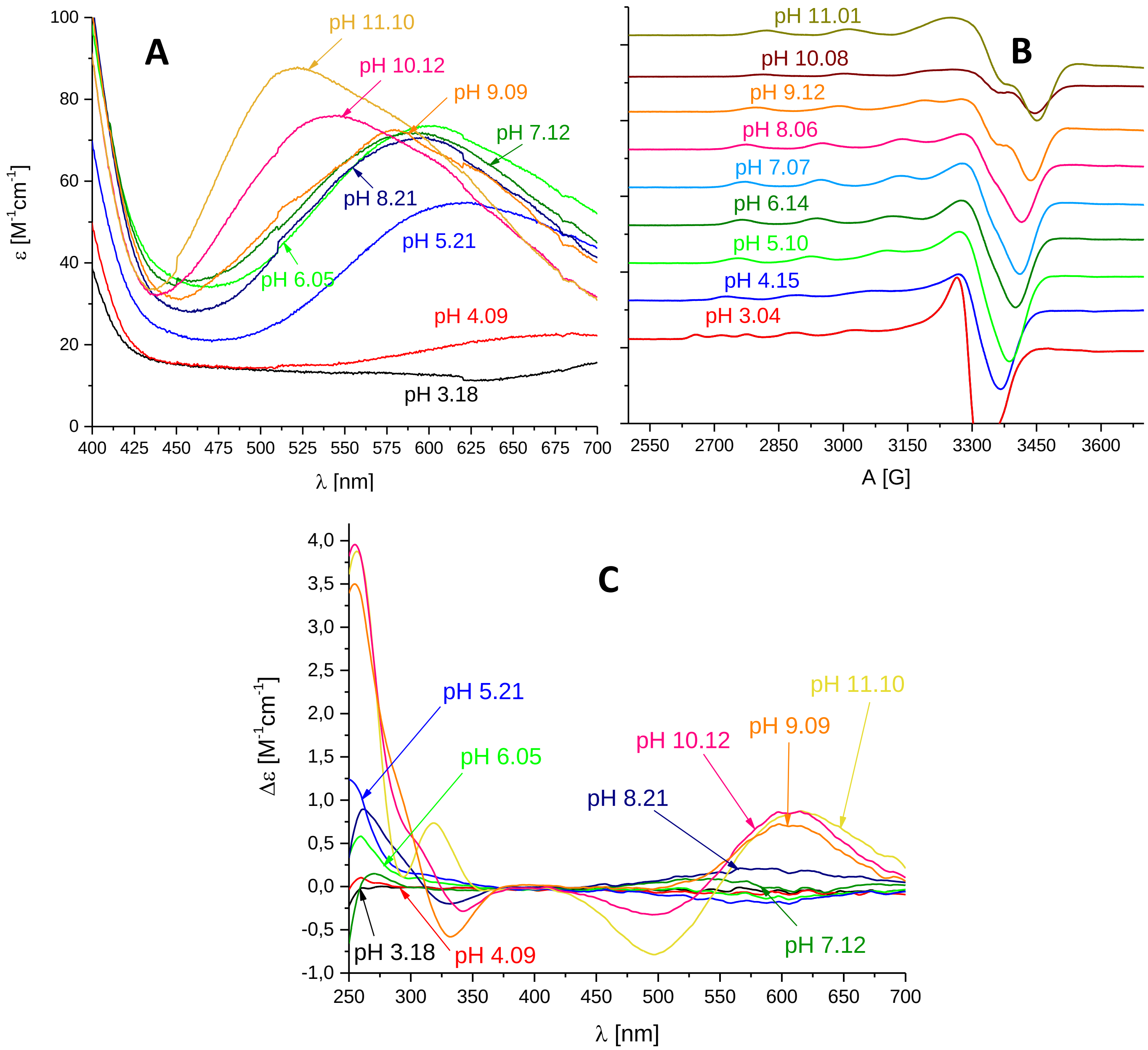

Figure S10. UV-Vis (A), EPR (B) and CD (D) spectra of Cu(II)-L6 system over the pH range 2–11. Conditions: T = 298 K and metal to ligand ratio = 0.8:1; [Cu(II)] = 4 × 10^{-4} M.

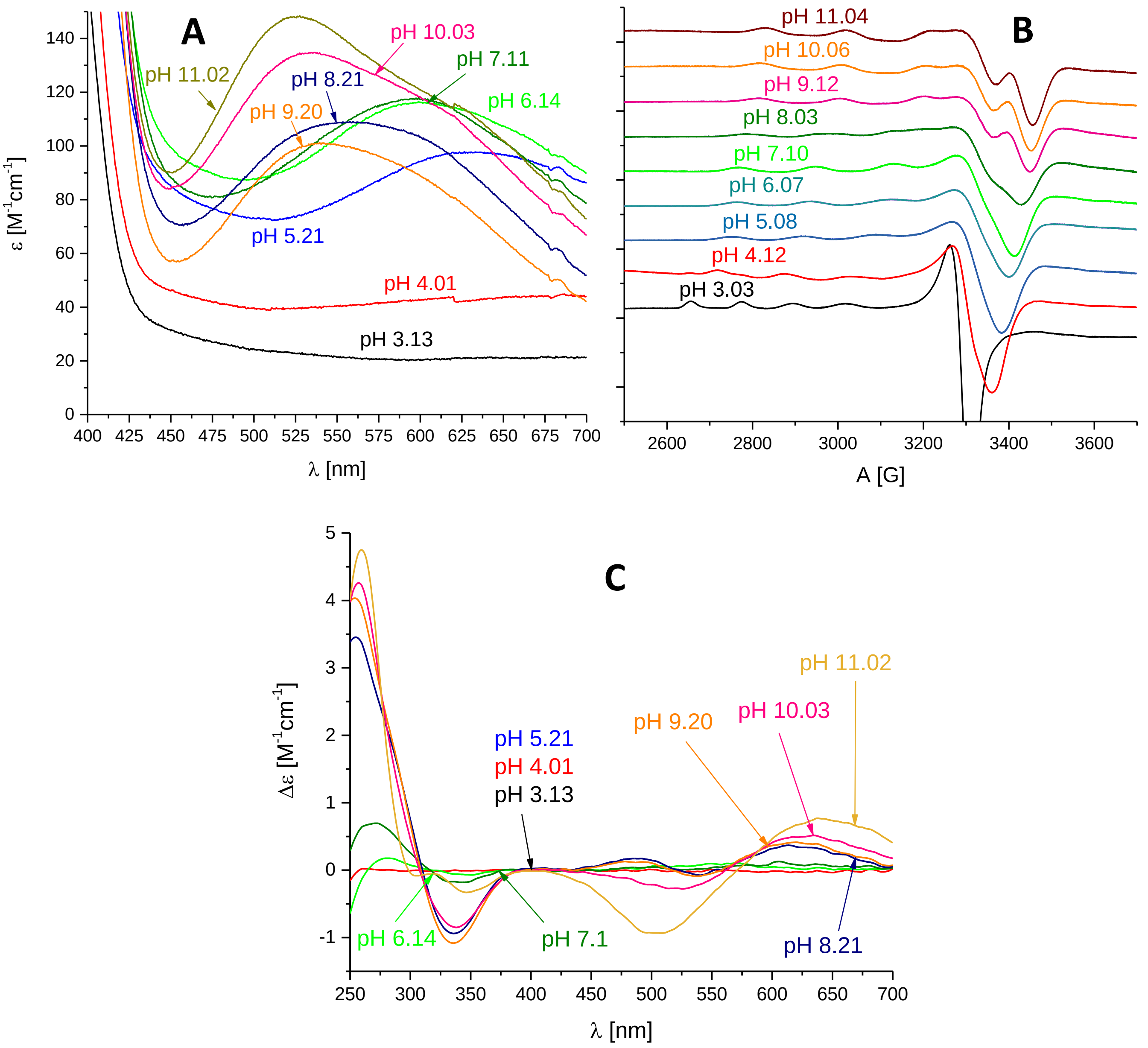

Figure S11. UV-Vis (A), EPR (B) and CD (D) spectra of Cu(II)-L7 system over the pH range 2–11. Conditions: T = 298 K and metal to ligand ratio = 0.8:1; [Cu(II)] = 4 × 10^{-4} M.

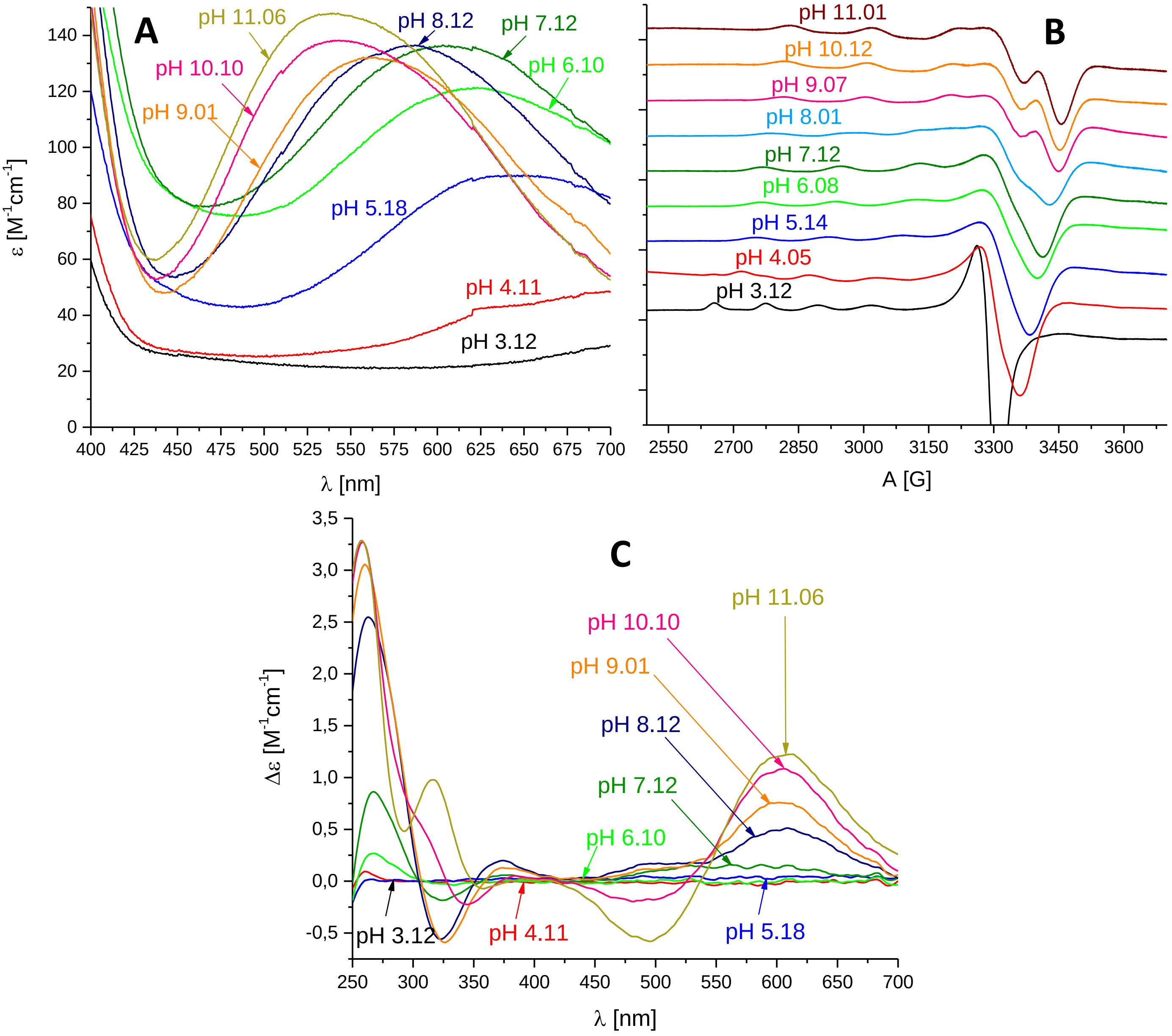

Figure S12. UV-Vis (A), EPR (B) and CD (D) spectra of Cu(II)-L8 system over the pH range 2–11. Conditions: T = 298 K and metal to ligand ratio = 0.8:1; [Cu(II)] = 4 × 10^{-4} M.

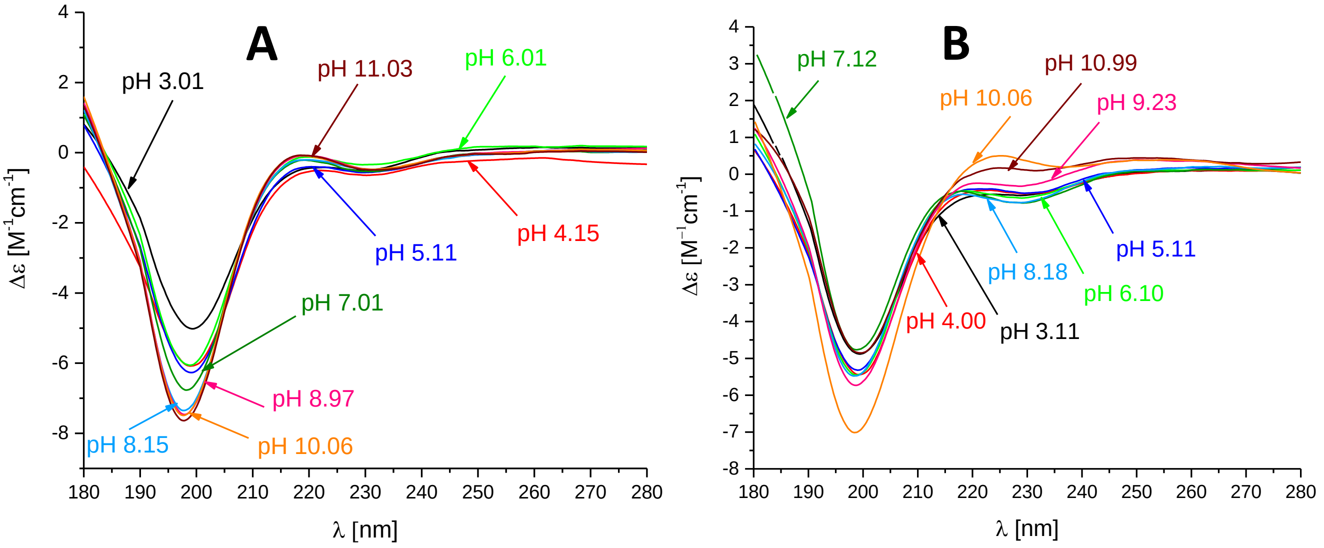

Figure S13. CD spectra of (A) L2: Ac-DKPAKAEDHDHHHGHAH peptide, (B) Cu(II)-L2 system over the pH range 2–11, 180-280 nm. Conditions: T = 298 K and metal to ligand ratio = 0.8:1; [Cu(II)] = 4 × 10^{-4} M.

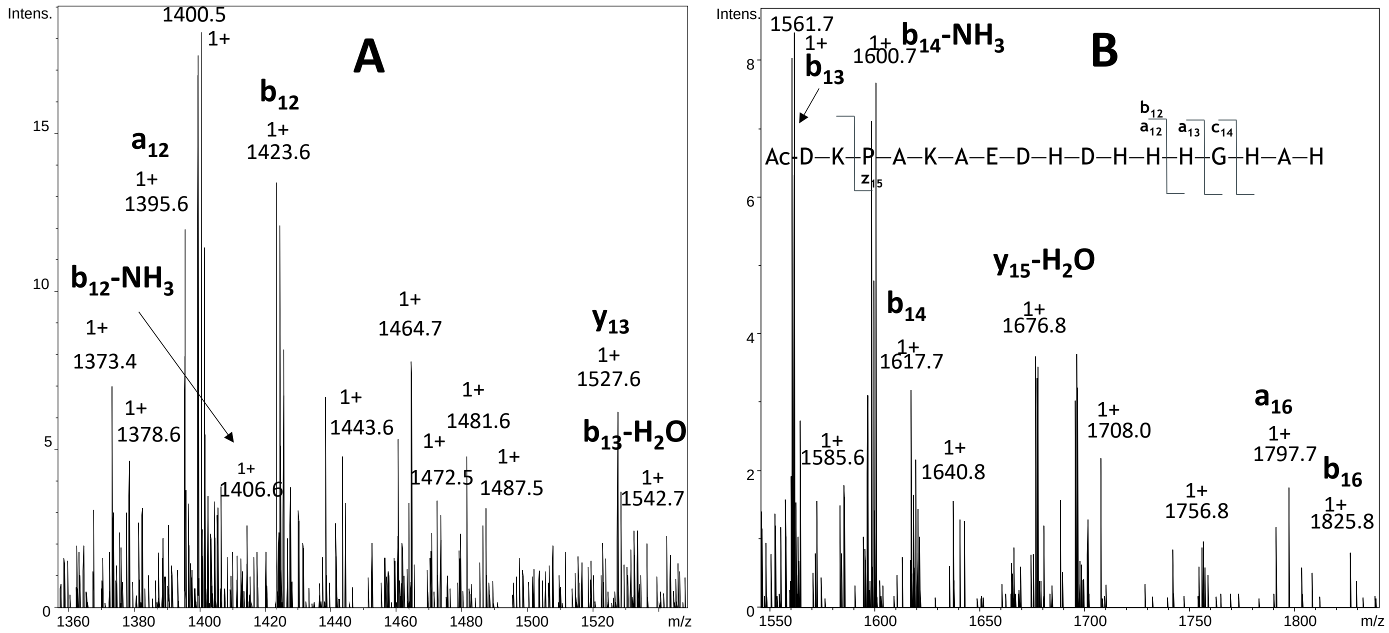

Figure S14. ESI-MS/MS spectrum of Cu(II)-L2 sample at (A) 1350-1575 *m/z* range, (B) 1550-1850 *m/z* range. Parent ion *m/z* = 991.44, z = 2+; collision energy 40 eV.
